# Rational Design for Monodisperse Gallium Nanoparticles by In Situ Monitoring with Small-Angle X-ray Scattering

**DOI:** 10.1021/jacs.5c00317

**Published:** 2025-03-26

**Authors:** Florian M. Schenk, Simon Wintersteller, Jasper Clarysse, Hanglin He, Jean-Marc von Mentlen, Nuri Yazdani, Markus Wied, Vanessa Wood, Christian Prehal, Maksym Yarema

**Affiliations:** † Chemistry and Materials Design Group, Institute for Electronics, Department of Information Technology and Electrical Engineering, ETH Zurich, Zurich CH-8092, Switzerland; ‡ Materials and Device Engineering Group, Institute for Electronics, Department of Information Technology and Electrical Engineering, ETH Zurich, Zurich CH-8092, Switzerland; § Department of Chemistry and Physics of Materials, Paris-Lodron-University of Salzburg, Salzburg AT-5020, Austria

## Abstract

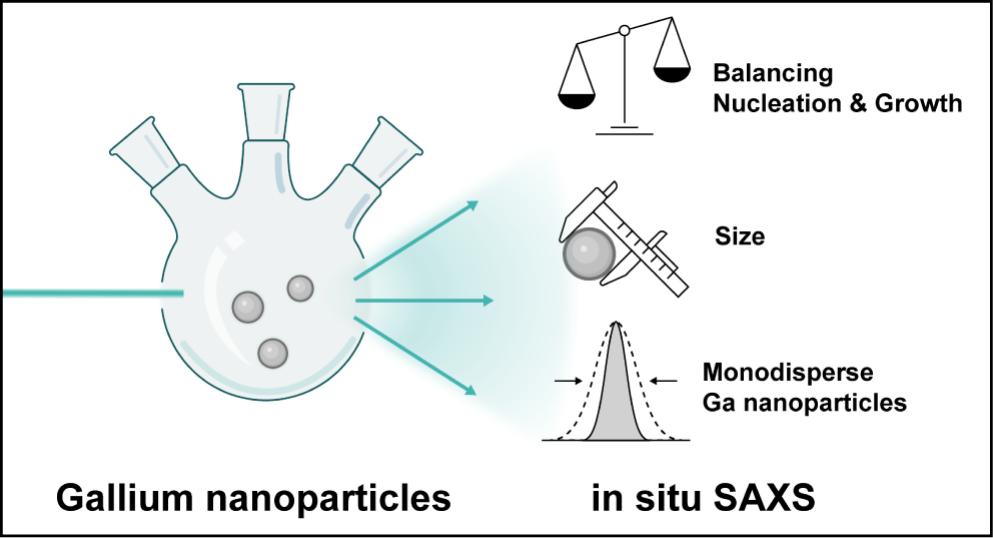

Colloidal chemistry is a well-known synthetic platform for producing size-uniform nanoparticles. However, the optimization of each material system still relies on a tedious trial-and-error approach in a multiparametric space, commonly referred to as design-of-experiments. This process is particularly laborious for emerging material classes for which only a handful of syntheses have been reported. Alternative approaches for the rational design of colloidal nanoparticles involve studying the reaction with in situ methods, thereby revealing the true underlying rules for the synthesis of monodisperse nanoparticles. Here, we focus on highly promising but little-studied colloidal gallium nanoparticles, using synchrotron-based small-angle X-ray scattering as a highly suitable in situ monitoring technique. We investigate the intertwined effects of process temperature, concentration of reactants, and the sterics of surface ligands during the hot-injection synthesis of gallium colloids. For quantitative comparison, we provide a description of gallium synthesis through the timestamps of partially overlapping reaction, nucleation, and growth stages. Our results reveal the key role of surface ligands in balancing the kinetics of nucleation and growth, as well as in enabling colloidal stability during the synthesis. Furthermore, we demonstrate that the large overlap between the nucleation and growth stages does not preclude the formation of monodisperse gallium nanoparticles. Our in situ experiments suggest several possible strategies for achieving size-uniform colloidal nanoparticles, thus enabling a rational design for the peculiar system of liquid metal nanodroplets and offering insights that can be extended to other monodisperse colloids prepared via hot-injection synthesis.

## Introduction

Liquid metals and alloys have sparked considerable interest in recent years across a variety of fields, such as catalysis,^1^ biomedicine,^2^ photonics^3^ as well as fundamental materials science.^4^ Research has focused on pure gallium and its alloys due to their low toxicity and stability through surface passivation via a native thin oxide shell.^5^ Gallium is a fascinating element with unique properties, including a bulk melting point close to room temperature (29.8 °C),^6^ anomalous volume expansion during freezing,^7^ and a complex phase diagram with several stable and metastable polymorphs.^7^ Liquid metals are highly attractive for a diverse set of catalytic applications due to their ability to dissolve catalytically active metals and their highly dynamic surface.^1^ This aids in overcoming typical limitations of heterogeneous catalysis, such as sintering or coke poisoning.^8^ In fact, 2 wt % copper in liquid gallium has shown increased catalytic productivity and improved stability in ammonia synthesis.^9^ In electronics, gallium enables liquid metal-based flexible electronics^10^ and self-healing anodes for lithium-ion batteries.^11^ In biology and medicine, their antimicrobial and antitumor properties, while maintaining low toxicity, are highly favorable.^2,12^

Gallium nanoparticles combine the unique benefits of liquid metals with nanoscale effects. For instance, they crystallize in the δ-phase at particularly low temperatures of 150 K, a result of strong melting point depression.^13^ Even under harsh catalytic conditions, such as 450 °C for alkane dehydrogenation, the high surface area, as well as activity and selectivity, are preserved.^8^ In electrocatalytic CO_2_ reduction, the nanoparticles are protected against coalescence by a thin oxide layer, preserving the high Faradaic efficiency (30%).^14^ Photonic applications benefit from the variable plasmonic properties of metallic gallium and its stability.^3^ The optical absorption of gallium nanoparticles varies between the liquid and solid state,^15^ and depends on the crystal phase,^16^ which makes it interesting for tunable photonic elements^15^ and optical phase change memory.^17^ The plasmonic response is also strongly size-dependent, with the peak wavelength ranging between 800 and 240 nm for particle sizes from 10 to 190 nm, as shown by EELS-STEM on a single-particle level.^18^

Most liquid metal nanoparticle syntheses are top-down approaches. Breaking up bulk liquid metals into nanoparticles either by ultrasonication^19^ or shear mixing^20^ are scalable and fast methods.^2^ The direct addition of surface ligands improves stability or functionalizes the surface liquid metal nanoparticles.^2^ While the average size can be tuned to some degree by variation of the temperature or through additives such as ligands or acids, these approaches typically result in broad size dispersions.^19^ In contrast, bottom-up approaches build liquid metal nanoparticles from the atomic scale, providing greater control over size and size dispersion, thereby enabling access to size-dependent properties. Colloidal synthesis offers a particularly cost-effective avenue to produce size-tunable and monodisperse nanoparticles.^21^ However, only a handful of syntheses exist for monodisperse liquid metal nanoparticles,^13^ and the understanding of these systems remains limited.

To monitor the formation of colloidal particles, time-resolved in situ techniques are exceptionally advantageous. With these methods, it is possible to track the evolution of nanomaterials during their synthesis, including size and polydispersity, and the chemical and structural changes of their respective precursors. Improved understanding of the elementary processes can aid in establishing guidelines for a targeted and more controlled synthesis of nanomaterials.^22^ For instance, in situ small-angle X-ray scattering (SAXS) has revealed classical and nonclassical nucleation mechanisms of gold and iron oxide nanoparticles, respectively.^23,24^ Similarly, using in situ SAXS, the stepwise growth of CsPbBr_3_ nanocrystals via aggregation of Cs[PbBr_3_] units has been observed.^25^ In CdSe quantum dots, it has been shown that a strong size dependency of the growth rate leads to absolute size focusing of the particle ensemble, despite extended nucleation during growth.^26^ In addition, the identity and time evolution of key reaction intermediates are crucial to the synthesis outcome. In this regard, X-ray absorption spectroscopy (XAS) and small-angle X-ray scattering have been used to track the formation and decomposition of copper phosphonate into copper nanospheres via lamella formation.^27^ In the case of Ir nanoparticles, the strong influence of the Ir precursor on intermediate metal species and their reaction mechanism was resolved via X-ray total scattering and pair distribution function (PDF) analysis.^28^

Here, we investigate the hot-injection synthesis^13^ of gallium nanoparticles via in situ small-angle X-ray scattering (SAXS). We select a previously reported method, which is based on the thermolysis of the tris(dimethylamido)gallium dimer at temperatures between 230 and 280 °C and in the presence of a secondary amine (e.g., dioctylamine or didodecylamine). Using in situ SAXS, we track the formation of gallium nanoparticles and derive timestamps of the elementary steps of precursor reaction, nucleation, and growth. Through combined fitting of form and structure factors, we quantify the structure of gallium products across length scales. For the primary structure (dispersed gallium nanoparticles), we extract the average diameter, polydispersity, yield, and the number of particles as the reaction progresses. We also investigate the secondary structure (agglomerated gallium nanoparticles) and quantify its percentage, volume fraction, and fractal dimension. Importantly, we reveal that the time overlap between nucleation and growth is significant for all reaction conditions. Despite this, a monodisperse ensemble of gallium nanoparticles can still be achieved. Our work provides a unique in situ perspective on hot-injection synthesis, detecting several critical reaction parameters beyond the design of burst nucleation. We elucidate the benefits of using a secondary amine surfactant and the role of kinetics in the nucleation and growth stages. Our in situ monitoring approach offers an efficient platform for the rational design of other monodisperse nanocrystals via hot-injection synthesis.

## Results and Discussion

### In situ SAXS Experimental Setup

**Figure 1:**
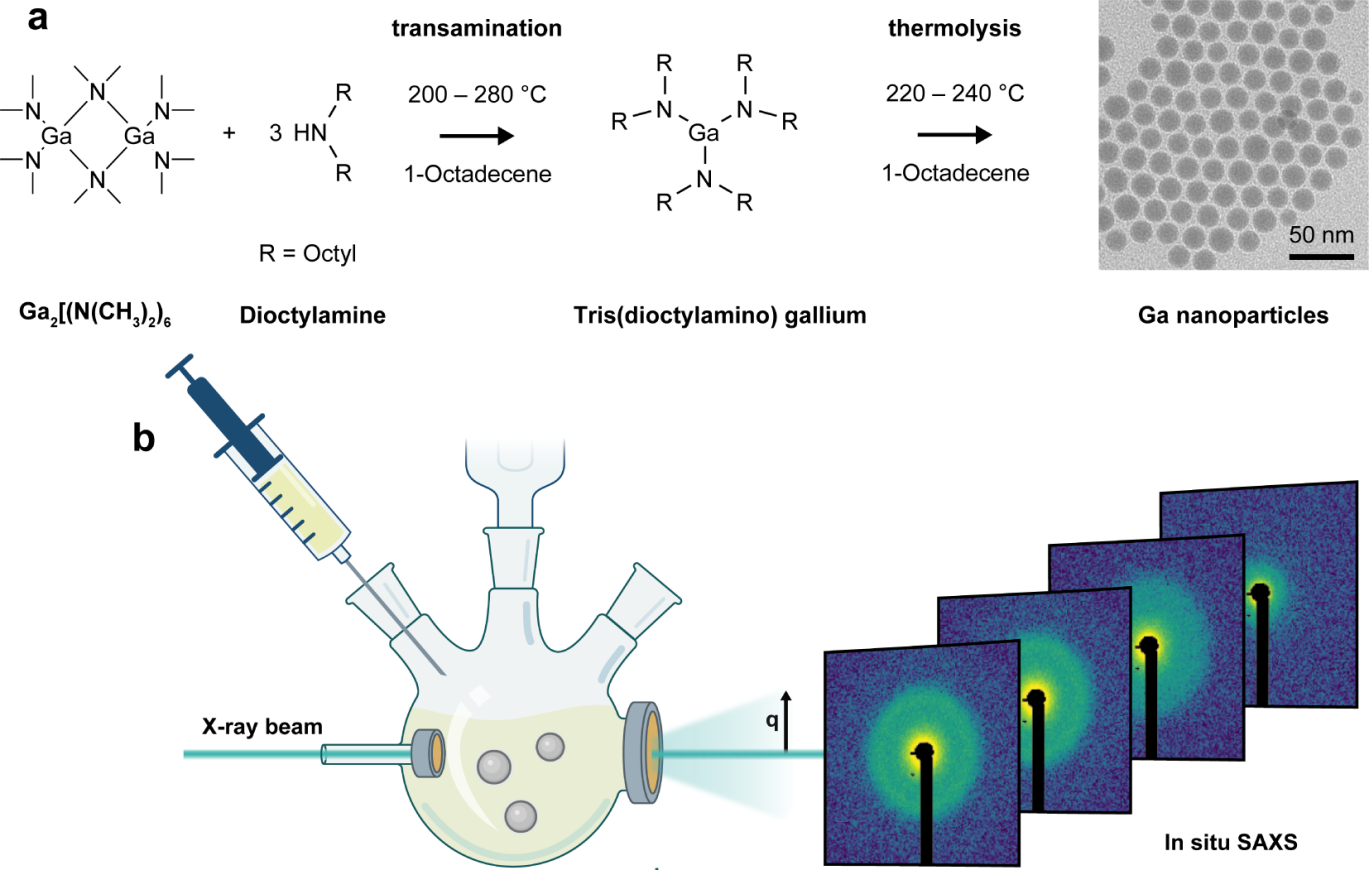
Synthesis of Ggallium nanoparticles, monitored by in situ small-angle X-ray scattering. (a) Synthesis scheme, illustrating the two reaction stages: transamination of the gallium precursor with a long-chain secondary alkylamine, followed by thermolysis and formation of gallium nanoparticles. (b) Schematic of the experimental setup. The in situ reactor is a tailor-made three-neck flask equipped with Kapton windows, magnetic stirrer, condenser, heating band, and automated syringe pump. During the entire synthesis, small-angle X-ray scattering can be recorded continuously.

We selected a previously reported synthesis of gallium nanoparticles for this study (Figure 1a).^13^ This approach involves the injection of a gallium amide precursor and a long-chain secondary amine, such as dioctylamine (DOA), into 1-octadecene (see Supporting Information for more synthetic details). The reaction begins with a transamination step between the dimeric tris(dimethylamido)gallium, Ga_2_[N(CH_3_)_2_]_6_, and a secondary amine. The resulting intermediate, gallium alkylamide, decomposes at elevated temperatures, leading to the nucleation and growth of colloidal gallium nanoparticles. Qualitative observations of the synthesis are as follows: the reaction solution turns pale yellow after the injection of the gallium precursor and remains so for tens of seconds into the reaction; afterward, the solution gradually becomes more and more brown while also becoming notably turbid, suggesting partial precipitation of gallium products (Figure S1). To understand the formation of monodisperse gallium nanodroplets, we conducted a set of synchrotron-based SAXS experiments. The experimental setup is shown schematically in Figure 1b. The custom-designed three-neck flask, equipped with two Kapton windows,^29^ enables high signal quality and time resolution. Importantly, we replicated synthetic conditions under the synchrotron beam, comparable to laboratory settings. Specifically, the reactor was kept stirring under a nitrogen atmosphere with the heating ramp and temperature profile remotely controlled. Finally, the reaction was initiated by the injection of the gallium precursor via a remotely controlled syringe pump (Figure S2). This setup enabled reproducible syntheses of gallium nanoparticles during which SAXS patterns could be continuously recorded. More experimental details are provided in the Supporting Information.

### Quantifying SAXS Data Sets

**Figure 2:**
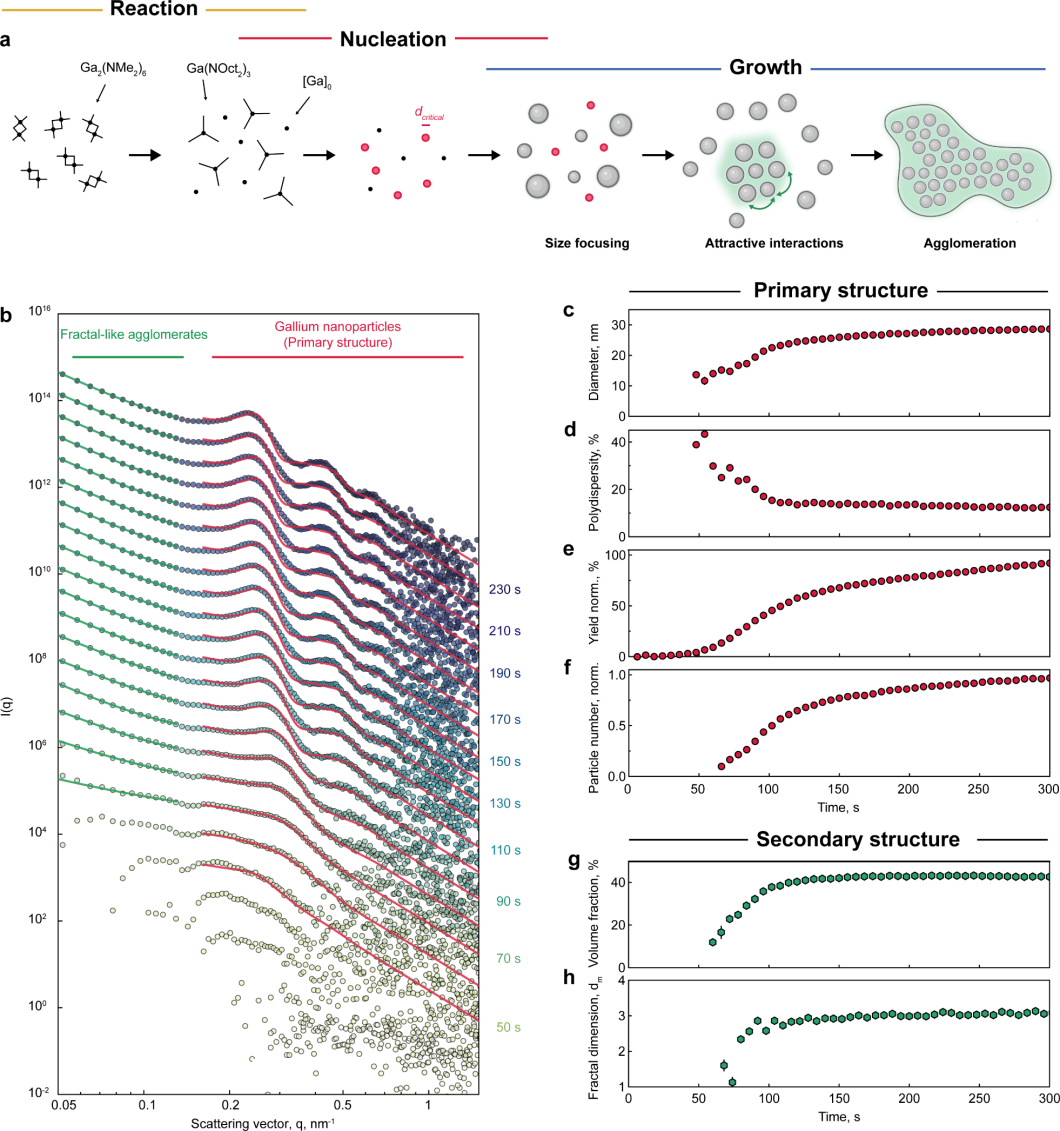
Fitting the in situ small-angle X-ray scattering curves. (a) Full description of the synthesis of gallium nanoparticles as derived from in situ SAXS measurements. The process includes reaction, nucleation, and growth as three stages, which overlap in time. Gallium nanoparticles are considered as a primary structure and agglomerates of gallium nanoparticles as a secondary structure. (b) Evolution of SAXS patterns (circles) during the synthesis of gallium nanoparticles (synthetic conditions: 0.37 mmol of Ga precursor, [DOA]:[Ga] molar ratio of 30, and a growth temperature of 230 °C). Red lines indicate the fitted function using a spherical form factor and a hard sphere structure factor. Green lines indicate the fitted function using a power law. The data are cascaded for better visibility. (c–h) Extracted parameters from in situ SAXS data fits in (b): average diameter of Gallium nanoparticles (c), relative polydispersity using Schulz–Zimm distribution (d), normalized reaction yield (e), normalized number of gallium particles (f), the volume fraction of Ga within the agglomerates (g), and the fractal dimension of the agglomerate structures (h).

SAXS curves contain extensive structural information, enabling the detection and quantification of scatterers across length scales. In situ SAXS measurements allow us to monitor the synthesis of gallium nanoparticles and develop a full description of the process, which can be formalized into three partially overlapping stages: the precursor reaction, nucleation, and growth (Figure 2a). Figure 2b shows a typical set of in situ SAXS patterns. The time onset (*t* = 0 s) is defined as the start of the gallium amide precursor injection. The synthesis typically progresses for 4 – 5 min. During the first few tens of seconds, no scattering or very weak scattering is observed. This corresponds to the transamination reaction step (Figure 1a), during which a primarily molecular conversion occurs. Next, at around 40–60 s into the reaction, the scattering intensity increases, generally featuring a plateau in the low-q region (<0.3 nm^–1^) and oscillations at higher scattering vectors. This suggests the formation of nanoparticles, staying in accordance with original synthesis.^13^ As the synthesis progresses further, the SAXS curves shift systematically to lower scattering vectors, manifesting the growth of the particle ensemble. Simultaneously, the minima in the oscillations become sharper, indicating a narrowing size dispersion. Notably, the low-q region deviates from the typical flat plateau, which would be expected for a dilute sample.^30^ Instead, an upward bend of the low-q plateau and a pronounced correlation peak at around 0.25 nm^–1^ become apparent over the course of the reaction (Figure 2b). We associate this with the formation of a larger secondary structure, such as an agglomeration of gallium nanoparticles. Thus, the in situ SAXS results remain in excellent agreement with visual observations of the synthesis (Figure S1).

To quantify the evolution of size and size dispersion of Gallium nanoparticles, we fit a spherical form factor with a Schulz–Zimm size distribution.^31^ While this simpler model fits the oscillation region very well, a hard sphere structure factor can be added to model the interaction between nanoparticles (red lines in Figure 2b). It is important to mention that the SAXS fits with and without a hard sphere structure factor show very similar quantitative trends for growing Gallium nanoparticles (Figures S3–S4). Finally, to assess the secondary structure, we fit the following power law^32^ to the low-q region (Figure 2b, green lines): , where *c* is a prefactor and *d*_m_ is a fractal dimension.^32^ This fitting methodology completes the picture for the formation of Gallium nanoparticles across length scales, including the primary structure (10–30 nm spheres) and their agglomerates (hundreds of nanometers). Further details for data evaluation, such as background correction and model fitting, are provided in the (Figures S3–S6).

Figure 2c–h shows the quantifications for the first 300 s of the synthesis, from which several structural parameters of growing gallium nanoparticles are extracted: (i) average diameter and relative polydispersity are obtained directly from spherical form factor fits and are indicative of growth and size focusing; (ii) normalized synthesis yield is calculated by integration of the scattering intensity between 0.05 and 2 nm^–1^ (Figure S7); and (iii) the number of nanoparticles is determined from the scale factor, normalized by the particle volume. Furthermore, we also evaluate the secondary structure by calculating the volume fraction of gallium in agglomerates and the dimensionality of such gallium assemblies.

Concerning the primary structure (i.e., dispersed colloidal nanoparticles), we can first obtain a reliable fit after approximately 1 min of synthesis time (Figure 2c,d,f). At this point, we already observe relatively large gallium nanoparticles (around 12 nm in diameter) with a broad size dispersion of about 30 – 40%. Despite this rather large size, the synthesis can still be considered in its initial stages, since both the yield and the number of particles are approximately 10–15% of their final values. This observation can be attributed to a large critical radius of gallium nanoparticles, stemming from a combination of low supersaturation values,^33^ high surface energy of liquid gallium (approximately 700 mJ/m^2^),^34^ and a large initial growth rate. A calculation of the size-dependent growth rate supports our reasoning regarding the large critical radius of gallium nanoparticles (Figure S8). During the second minute of synthesis, gallium nanoparticles grow rapidly to >20 nm, while their relative polydispersity decreases notably to around 12% (Figure 2c,d). This size-focusing phenomenon can be explained by the strong size dependency of the growth (Figure S8), possibly due to the lowering of intrinsic surface reactivity or denser ligand coverage for larger sizes,^35,36^ both of which result in plummeting growth rates as size increases. After 2 min, gallium nanoparticles experience a considerably slower increase in the number of scatterers. This indicates the conclusion of the nucleation stage, during which nanoparticles continue to grow to approximately 28 nm and double in synthesis yield, while maintaining a low polydispersity of approximately 12% is preserved. Interestingly, the polydispersity does not deteriorate even with extended synthesis times of up to 1 h, indicating a negligibly slow mass transfer (i.e., Ostwald ripening) between liquid gallium nanoparticles (Figure S9). Such suppressed Ostwald ripening can facilitate straightforward upscaling of hot-injection syntheses,^37^ maximizing yield while preserving a narrow size distribution, which is preferred for advanced characterization and applications of liquid metal nanoparticles.

In parallel, the secondary structure (i.e., agglomerations of gallium nanoparticles) also evolves with time. Similarly, it is first observed after 1 min of synthesis time. Initially, loose agglomerates are formed, marked by the low volume fraction of gallium and the low fractal dimension around 1–2 (Figure 2g,h). This dimensionality factor suggests the formation of chain-like and plate-like agglomerate geometries (Figure S10). Subsequently, the secondary structure undergoes densification, as indicated by an increased volume fraction of up to 45% and a fractal dimension around 3 toward the end of the reaction. A fractal dimension of 3 is expected for three-dimensional agglomerates and has been observed before for agglomerated iron oxide particles.^32^ This agglomeration can also be observed visually during the synthesis, as the reaction solution gradually becomes turbid (Figure S1). Note that these secondary structures are randomly packed and do not show a strong long-range order. We therefore hypothesize that the initially formed small particles are colloidally stable and then destabilize during growth when the attractive interactions of the cores outweigh the repulsive interactions of the ligands. Agglomeration and formation of superstructures have been observed previously in some systems, such as in Au,^38^ Pd,^39^ and Fe–Co colloids.^40^ In the latter case, this facilitates the formation of highly monodisperse batches of nanocrystals.

To summarize, the in situ SAXS measurements provide a full description of gallium synthesis (Figure 2a). After the initial transamination reaction stage, the intermediate gallium alkylamide undergoes thermolysis,^13^ which appears to be the rate-limiting step, as the synthesis requires a certain induction time before particle formation can be observed (Figure S1). This suggests that the system needs time to build up the necessary [Ga]_0_ concentration and therefore reach the critical supersaturation value. Subsequently, the nucleation stage begins, and gallium nanoparticles grow to their critical diameter, beyond which they enter a size-focusing regime and agglomeration of large gallium nanoparticles occurs. We anticipate that the reaction, nucleation, and growth stages of the process significantly overlap in time, affecting the quality of the obtained gallium nanoparticles. In order to understand these effects and to enable a rational design for the synthesis, we conduct a parametric study, tuning the growth temperature, concentration of amine, and length of the alkyl chain, while taking and quantifying in situ SAXS patterns as explained above (Figure 2b). To efficiently compare synthesis conditions, we extract the duration of the reaction, nucleation, and growth stages, as well as kinetic parameters such as reaction rate and nucleation rate.

### Effect of Temperature

**Figure 3:**
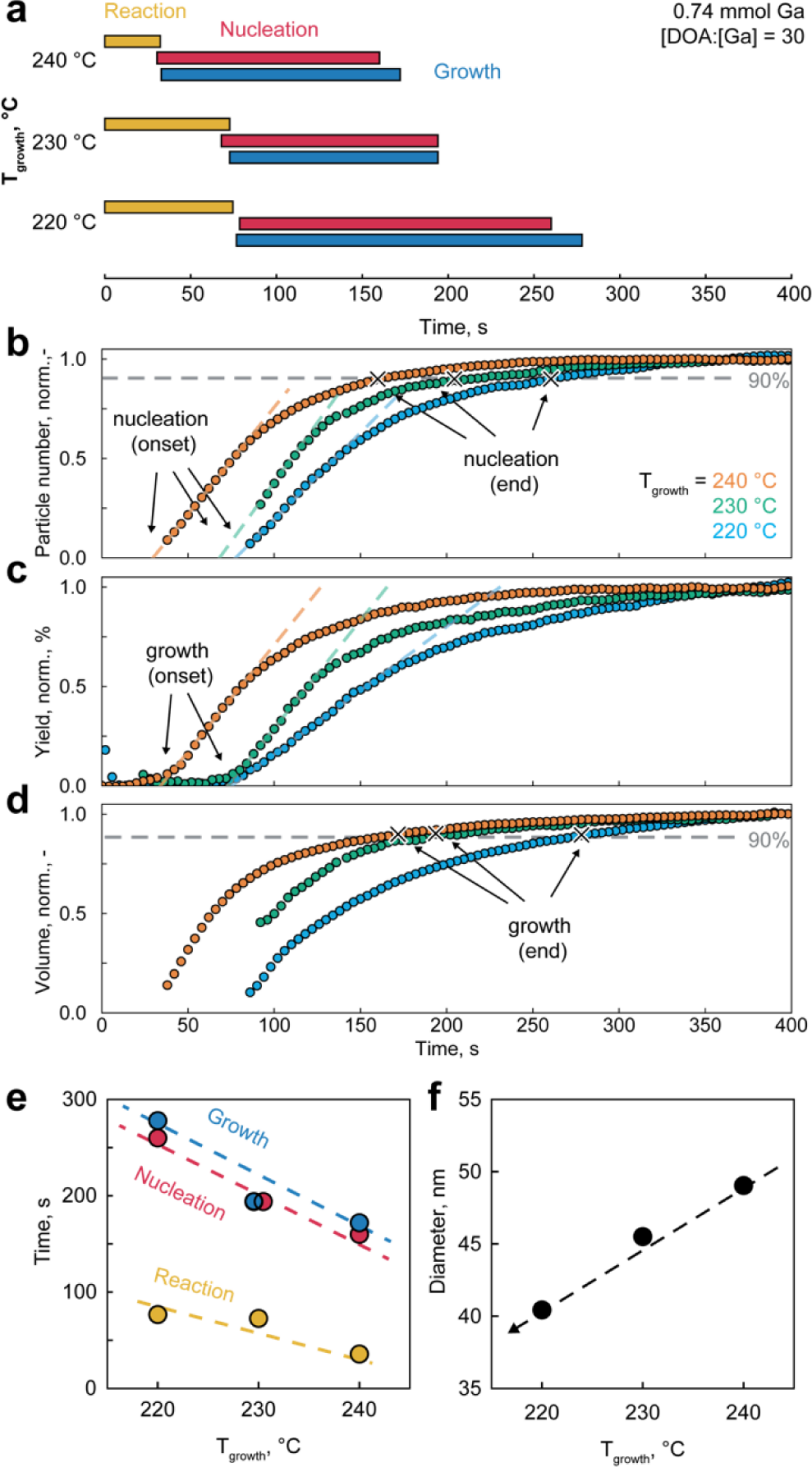
Tuning the growth temperature of gallium nanoparticle synthesis. (a) Duration of reaction, nucleation, and growth stages for the syntheses, carried out at 220, 230, and 240 °C (all other conditions are kept constant: 0.74 mmol of Ga precursor, [DOA]:[Ga] molar ratio of 30). The characteristic times of the three stages are extracted from SAXS fittings as follow: (b) onset and end of the nucleation stage as 0 and 90% of the normalized number of gallium particles, (c) onset of the growth stage and end of the reaction stage as 0% of the normalized reaction yield, and (d) end of the growth stage as 90% of the normalized total volume of gallium nanoparticles. (e) The duration of the three stages and (f) the size of gallium nanoparticles for the syntheses at different growth temperatures. Data are color coded for growth temperature of 240 °C (in orange), 230 °C (in green), and 240 °C (in blue).

Figure 3a shows the timestamps of the reaction, nucleation, and growth stages for the syntheses carried out at three different temperatures. The start of the nucleation stage can be estimated as the onset of the normalized particle number (Figure 3b) by extrapolating the linear region of the plots to 0. The slope of the curves relates to the intrinsic nucleation kinetics of the process. We consider the end of nucleation to occur when the particle number reaches 90% of its final value. Similarly, the start of the growth stage and its kinetics can be extracted as the onset and slope of the normalized yield, respectively (Figure 3c). Finally, the end of the growth stage can be assumed to occur when the total volume of gallium nanoparticles reaches 90% of its final value (Figure 3d). We noted that the nucleation and growth stages often start almost simultaneously (or within the error bar of the linear extrapolation, Figure S11), suggesting that the synthesis of gallium nanoparticles is a growth-dominated process from the viewpoint of classical nucleation theory. We further modeled the reaction as first-order kinetic processes, resulting in closely similar relations for nucleation and growth timestamps (Figure S12). It is important to mention that in situ SAXS cannot capture the precursor reaction stage and the formation of [Ga]_0_ monomers. Therefore, for consistency, we assume that the reaction stage starts with the injection time (*t* = 0 s) and ends when the growth stage begins.

As expected, all stages show accelerated kinetics with increasing temperature, demonstrating a strong trend despite the narrow temperature window. For instance, the onset of particle formation requires around 70 s at 220 °C, but only 35 s at 240 °C. This is further illustrated in Figure 3e, where the endpoints of the reaction, nucleation, and growth stages are plotted as functions of the growth temperature. While all processes accelerate with increasing temperature, the nucleation and growth stages have a stronger temperature dependence than that of the precursor reaction. Consequently, larger gallium nanoparticles are formed at higher growth temperatures (Figure 3f), providing a convenient tool for tuning particle size. In further agreement with the rate law, lower concentrations of the gallium amide precursor result in smaller gallium nanoparticles (Figure S13). Similar kinetic effects have been reported for many metallic systems, such as Bi, In, or Sn nanoparticles.^41−43^

Finally, we note that the nucleation and growth processes overlap almost entirely (Figure 3a). Despite this, the final polydispersity of gallium nanoparticles remains low, ranging between 12% and 20%. We will quantify this overlap and discuss it in the later sections of the paper.

### Effects of Secondary Amine

We investigate two effects stemming from the secondary amine in gallium synthesis. First, we tune the amount of dioctylamine with respect to the gallium precursor. Afterward, we compare dioctylamine with didodecylamine (Figure S14), thus revealing the influence of different alkyl chain lengths.

**Figure 4:**
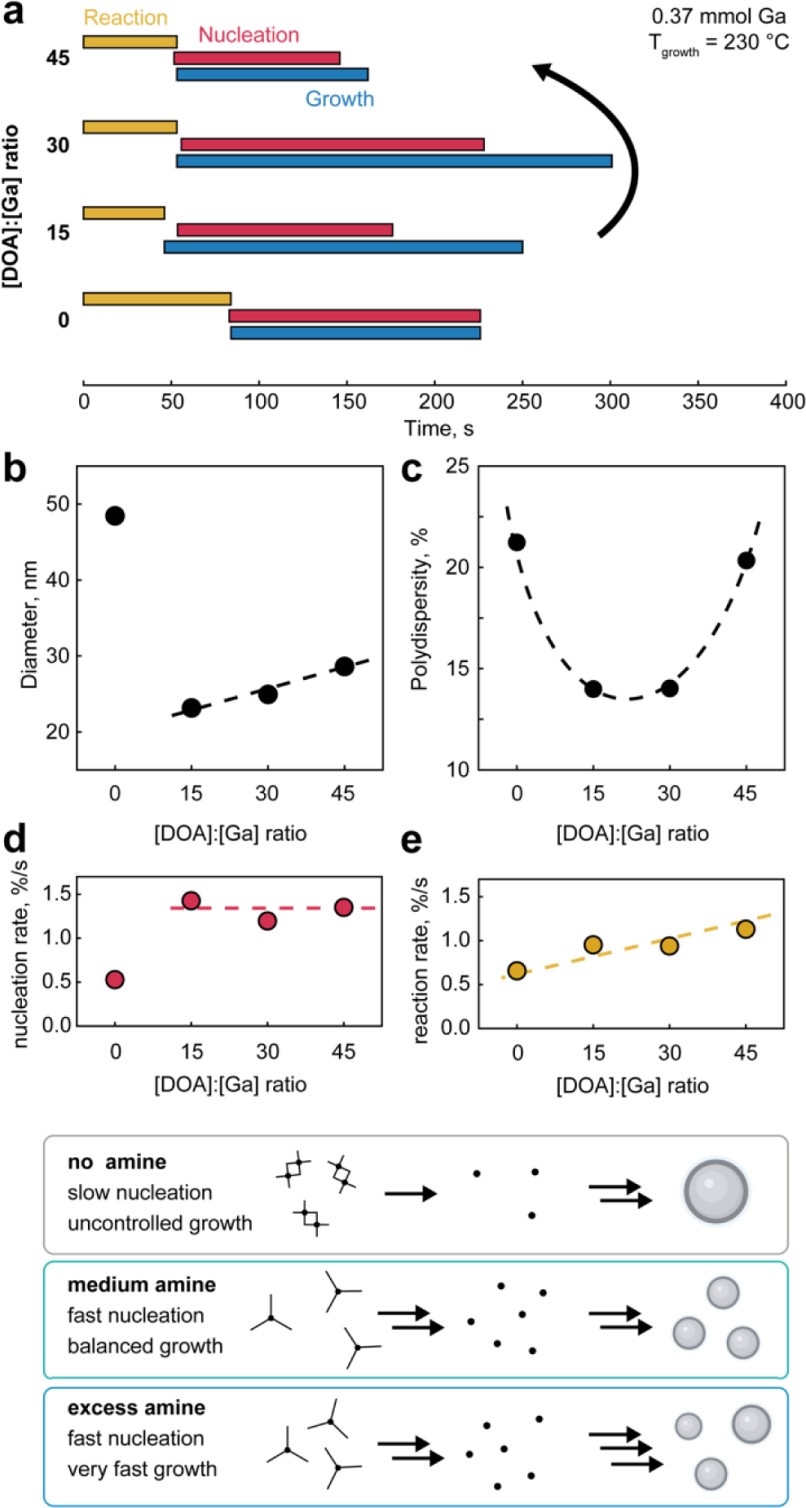
Tuning the amount of secondary amine. (a) Timestamps of reaction, nucleation and growth stages for the syntheses, carried out with various amount of dioctylamine (all other conditions are kept constant: 0.37 mmol of Ga precursor, *T*_growth_ of 230 °C). (b–e) Effects of different dioctylamine-to-Ga-precursor molar ratios on (b) size, (c) size distribution, (d) nucleation rate, and (e) growth rate of gallium nanoparticles. (f) Schematic illustration of the role of secondary amine on relative kinetics of nucleation and growth stages of the synthesis.

Tuning the ratio between dioctylamine and gallium amide, we observe interesting nonlinear trends (Figure 4a). In the absence of any secondary amine, the reaction is slower, which delays nucleation and growth onsets to approximately 70–75 s of synthesis time. We associate this observation with the stability of the initial gallium amide precursor; i.e., the absence of secondary amine in the mixture excludes the transamination step, thereby delaying the formation of [Ga]_0_ monomers. Furthermore, the growth of gallium nanoparticles becomes uncontrollable due to the lack of ligands, leading to very large sizes of gallium nanoparticles (up to 50 nm, Figure 4b) and high polydispersity values (>20%, Figure 4c).

Adding dioctylamine improves the quality of the gallium nanoparticles. This is particularly evident for dioctylamine-to-gallium ratios of 15 and 30: the size of gallium nanoparticles is much smaller, and the size distribution is reduced to 14% (Figure 4b,c). Under these conditions, we observe a shorter reaction stage, indicating the formation of more reactive gallium alkylamides. Extracting nucleation rates provides a clear distinction between the kinetics of the initial gallium precursor and the more reactive intermediate alkylamide (Figure 4d). The growth stage, however, is longer in the presence of the secondary amine, as their alkyl chains create steric hindrance on the surface of the growing nanoparticles (Figure 4a).

Further increasing the amount of dioctylamine results in a growing imbalance in synthesis kinetics. At a [DOA]:[Ga] ratio of 45, the nucleation and growth stages are both shorter, leading to larger gallium nanoparticles and broader size distributions (Figure 4a–c). We associate these observations with the excessive amount of dioctylamine in the flask, as the secondary amines may promote the synthesis by acting as reducing agent for the Ga precursor. This is supported by a linear increase in gallium size with the amount of dioctylamine (Figure 4b) and by extracted reaction rates, which we relate to the growth of gallium nanoparticles from [Ga]_0_ monomers (Figure 4e).

The influence of the dioctylamine amount is summarized schematically in Figure 4f. In the absence of a secondary amine, the nucleation of gallium seeds is slow, while the growth is uncontrolled due to the lack of surface passivation. The presence of dioctylamine provides a boost to the nucleation rate, while the growth rate is also improved proportionally to the amount of secondary amine. This leads to an optimum, where medium amounts of dioctylamine facilitate fast nucleation and matching growth kinetics, resulting in gallium nanoparticles with improved size distributions.

**Figure 5:**
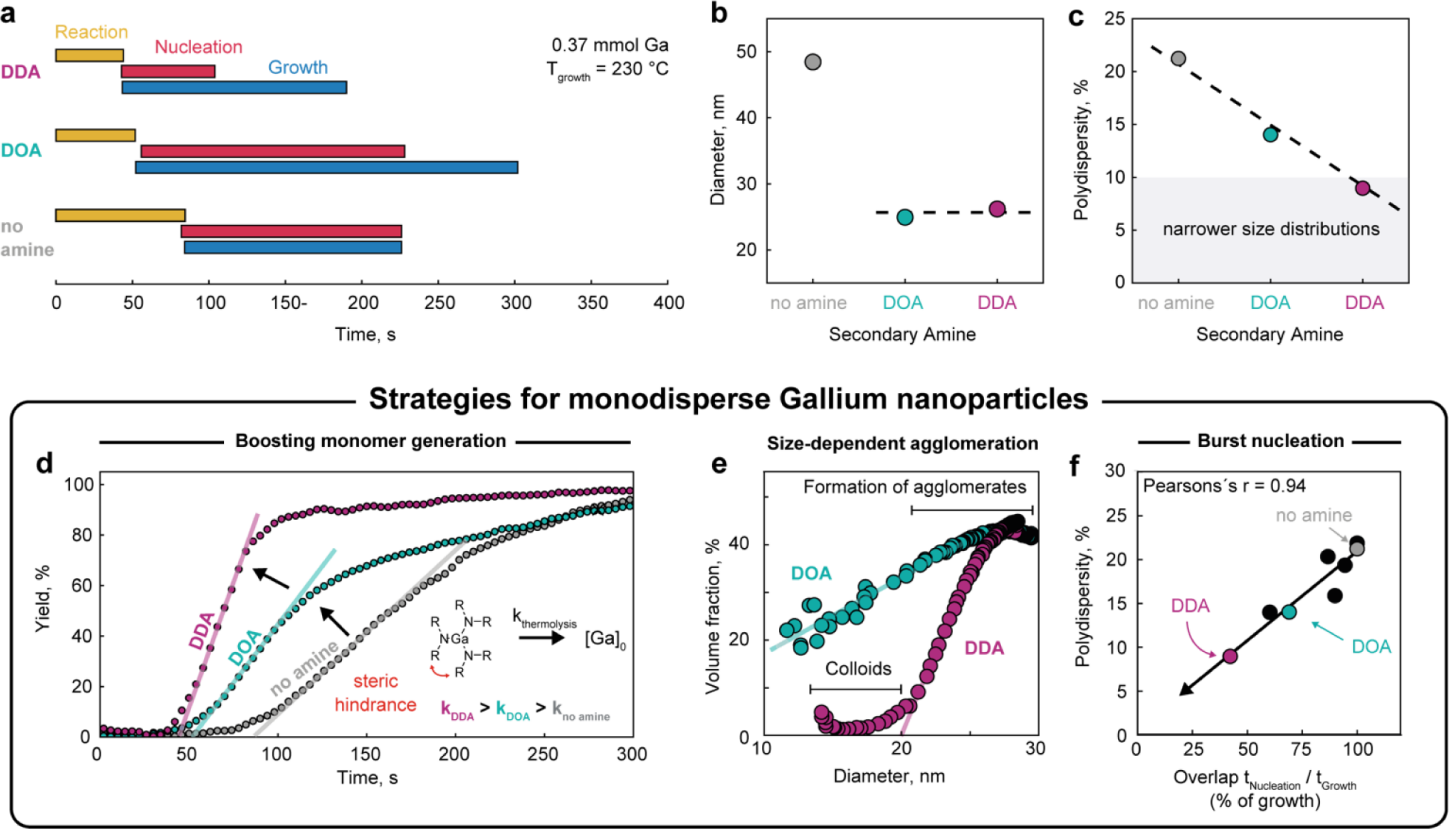
Reaching monodisperse gallium nanoparticles. (a) Timestamps of reaction, nucleation and growth stages for the syntheses, carried out with different secondary amines and without an amine (all other conditions are kept constant: 0.37 mmol of Ga precursor, *T*_growth_ of 230 °C). (b,c) Size and size distribution of gallium nanoparticles, prepared with and without a secondary amines (DOA is dioctylamine and DDA is didodecylamine). (d–f) Triune strategies for monodisperse gallium nanoparticles: (d) Normalized reaction yield of gallium nanoparticles, showing improved growth kinetics in the presence of long-chain alkylamines; (e) volume fraction of the agglomerated particles as a function of the gallium diameter, showing improved colloidal stability for didodecylamine; and (f) correlation of polydispersity and the temporal overlap between the nucleation and growth stages, showing more effective separation of nucleation and growth for didodecylamine. Data are color-coded for didodecylamine (pearly purple) and for dioctylamine (in turquoise).

We borrow these optimal conditions and replace dioctylamine with longer-chain didodecylamine in the synthesis mixture. Figure 5a shows a timestamp of the syntheses with two secondary amines compared to the reaction without any amine in the mixture. For didodecylamine, all stages of the synthesis appear to occur faster with respect to dioctylamine. Didodecylamine molecules are more efficient in transamination and thermolysis, which translates into faster nucleation and growth processes. Interestingly, this also results in a smaller time overlap between the nucleation and growth stages. While the two secondary amines lead to similar final nanoparticle sizes (dictated by the amount of gallium precursor and growth temperature), the polydispersity for didodecylamine is clearly improved, reaching below the threshold of 10% for a narrow size distribution (Figure 5b,c).

### Strategies for Monodisperse Gallium Nanoparticles

We now consolidate all in situ SAXS data sets (Figures S15 and S16 and Table S2) and suggest several approaches to synthesize monodisperse batches of gallium nanoparticles. The first strategy relies on boosting the overall reaction of gallium precursors to the nanoparticles. To illustrate this, we plot the synthesis yield as a function of time for didodecylamine, dioctylamine, and no amine in the flask (Figure 5d). The fastest gallium formation is observed for didodecylamine, while the thermolysis rate with dioctylamine is twice as slow, yet still notably faster than when no secondary amine is present in the synthesis mixture. We attribute this result to steric hindrance, which arises between the long alkyl chains of amine ligands. Therefore, the bulkier didodecylamine forms a more labile intermediate dialkylamine complex, resulting in faster generation of [Ga]_0_ monomers. FTIR measurements suggest a possible reaction mechanism through the oxidation of secondary amine to aldimine and the formation of a short-lived gallium hydride complex (Figures S17 and S18). The accelerated thermolysis kinetics may facilitate reaching higher supersaturation values of [Ga]_0_ monomers, leading to a shorter nucleation stage, followed by the extended growth of monodisperse gallium nanoparticles. To test this hypothesis, we performed syntheses with a shorter secondary amine, dihexylamine, and investigated the Ga products. As expected, using dihexylamine results in slightly larger size and wider size dispersion (33 nm and 18%), which fall perfectly in line between no amine and dioctylamine (Figure S19).

The second strategy highlights the importance of colloidal stability and the agglomeration of growing nanoparticles. Figure 5e plots the volume fraction of gallium in the secondary structure as a function of nanoparticle size. For dioctylamine, the volume fraction is significant for all sizes, suggesting a simultaneous formation of agglomerates since the inception of the growth stage. In contrast, the volume fraction of gallium for the synthesis with didodecylamine is a step function, which remains negligible for particles smaller than 20 nm and rapidly increases above this threshold. We attribute this to the sufficiently long hydrocarbon chains of didodecylamine, which provide stronger repulsive forces between the nanoparticles and, thus, their colloidal stability up to sizes of 20 nm. We also noted that the agglomeration of gallium nanoparticles is partially size-selective, as manifested by the steepness of the volume fraction plot (Figure 5e). For larger sizes (above 27 nm), the volume fraction reaches approximately 40%, meaning that the agglomerates of gallium nanoparticles are compact and three-dimensional (Figure S10), cutting off monomer supply and halting growth. Similar effects of size-dependent colloidal stability, as well as the formation of superlattices during colloidal synthesis, have been demonstrated for Au,^38^ Pt,^39^ CoPt,^40^ CbPbBr_3_,^25^ and Fe–Co^40^ nanocrystals.

The third strategy underscores the importance of minimizing the time overlap between the nucleation and growth stages. Critically, we reveal a universal trend between the final polydispersity of gallium nanoparticles and the temporal overlap of nucleation and growth stages (expressed as a percentage of growth, Figure 5f). This dependence shows a high Pearson correlation coefficient of 0.95, regardless of reaction variables such as the concentration of gallium precursor and secondary amine, type of amine, and growth temperature. We conclude that controlled growth with respect to a fast nucleation stage can be achieved through a combination of several reaction parameters, namely, a relatively high growth temperature (Figure 3) and an optimal amount of secondary amine (Figure 4). The use of didodecylamine provides a further reduction in the temporal nucleation/growth overlap and, hence, the narrowest size distribution of Gallium nanoparticles (Figure 5). Despite this, even in didodecylamine-based synthesis, the overlap between nucleation and growth is still relatively large, approximately 45%. This contradicts the LaMer model (1950),^44^ which anticipates a complete temporal separation of nucleation and growth events for forming a monodisperse batch of nanocrystals.^33,45^ Today, this classical LaMer model remains under debate,^22^ since it has been shown that good size dispersion can be achieved in systems with strong overlap of nucleation and growth, such as in CdSe,^26^ InP,^46^ PbS,^47^ or Ir.^48^ Many nonclassical nucleation theory approaches have been discussed recently.^24,49^ For example, in several metallic nanoparticles, such as Ir,^50^ Rh, or Pd, the strong overlap of nucleation and growth can be explained by autocatalytic growth. While the nucleation is relatively slow, the fast growth is catalyzed by the nanoparticle itself.^48^ The synthesis of gallium nanoparticles is similar, and liquid metal gallium is widely regarded for its excellent catalytic properties.^1^

## Conclusions

In this work, the hot-injection synthesis of liquid gallium nanoparticles was investigated by in situ small-angle X-ray scattering. We obtained a full depiction of the evolution of gallium nanoparticles, extracting critical parameters such as size, polydispersity, synthesis yield, particle number, and morphology of agglomerates. Thereby, we tracked the particles from the inception of the nucleation stage until extended growth periods. By varying the synthesis parameters, such as the amount or type of secondary amine, the elementary synthesis steps could be monitored and optimized. The synthesis of Ga colloids is outstanding in that it features the delayed formation of nanoparticles, significant overlap between the nucleation and growth stages, and particle agglomeration during their growth. Still, monodisperse nanoparticles are formed due to the dual role of a secondary amine: (i) as a reactant in the formation of the labile intermediate Ga-complex, boosting production of Ga monomers, and (ii) as a surface ligand on the nanoparticles, controlling the size-selective agglomeration of gallium nanoparticles. Tuning the Ga-to-amine ratio in the reaction mixture allows nucleation and growth stages to be kinetically balanced, leading to improved size dispersion. Replacing dioctylamine with longer didodecylamine leads to several positive effects, including promoted nucleation and a narrow size dispersion of approximately 9%. Based on our in situ SAXS quantifications, we identified three design strategies to synergistically yield monodisperse gallium nanoparticles.

Our in situ methodology provides direct insights into the formation of liquid gallium nanoparticles and the influence of various synthetic parameters, which are valuable for understanding and advancing the controlled synthesis of liquid metal particles and their alloys. We anticipate further improvements in gallium synthesis by boosting the nucleation stage while controlling the growth stage, which can be achieved by introducing a reducing agent,^14^ while independently optimizing the surface ligand. Furthermore, our findings for gallium align with those observed in noble metal nanoparticles featuring autocatalytic growth (e.g., Ir, Rh, or Pd), with overlap of nucleation during growth and ligand-induced size focusing.^38^ In a broader perspective, we highlight the importance of in situ techniques to study the complex systems of colloidal nanocrystal synthesis. Specifically, in situ SAXS monitoring provides a distinct view of the nucleation and growth stages in addition to the underlying synthesis mechanism, facilitating the development of precise synthetic protocols. The precursor conversion (reaction) stage may be assessed through a combination of ex situ techniques (e.g., NMR or headspace gas chromatography)^51,52^ and simulation approaches (e.g., population balance modeling, PBM).^53−55^ Particularly in the case of gallium synthesis, we anticipate an interesting prenucleation behavior^24^ due to its rich cluster chemistry and strong tendency for Ga–Ga bond formation.^56,57^ We hope that our work motivates researchers to study emerging nanomaterial systems such as liquid metal gallium nanoparticles.

## Supporting Information Available

Synthetic details, detailed description of data analysis, supporting Figures S1–S19 and Tables S1–S2 (PDF)
