# Supplementary material for: Rational Design for Monodisperse Gallium Nanoparticles by In Situ Monitoring with Small-Angle X-ray Scattering
Source: J Am Chem Soc. 2025 Mar 26;147(14):12105–14. doi: 10.1021/jacs.5c00317 (PMC11987021; doi:10.1021/jacs.5c00317)
Supplement: Supplementary file 1 — ja5c00317_si_001.pdf [file ja5c00317_si_001.pdf]

Supporting Information

Rational Design for Monodisperse Gallium Nanoparticles by In Situ Monitoring with Small Angle X-ray Scattering

Florian M. Schenk,[1] Simon Wintersteller,[1] Jasper Clarysse,[2] Hanglin He,[1] Jean-Marc von Mentlen,[2] Nuri Yazdani,[2] Markus Wied,[2] Vanessa Wood,[2] Christian Prehal,[2,3] and Maksym Yarema[1,*]

[1] Chemistry and Materials Design Group, Institute for Electronics, Department of Information Technology and Electrical Engineering, ETH Zurich, CH-8092 Zurich, Switzerland

[2] Materials and Device Engineering Group, Institute for Electronics, Department of Information Technology and Electrical Engineering, ETH Zurich, CH-8092 Zurich, Switzerland

[3] Department of Chemistry and Physics of Materials, Paris-Lodron University of Salzburg, AT-5020 Salzburg, Austria

yaremam@ethz.ch

Materials and Methods:

Materials. Gallium tris(dimethylamido) dimer ($Ga_2(DMA)_6$, 98%) was purchased from American Elements and used as received. Dioctylamine (DOA, 98%), didodecylamine (DDA, >97%), dihexylamine (DHA, 97%), and 1-octadecene (1-ODE, technical, 90%) were bought from Sigma-Aldrich. DOA, DHA, DDA and 1-ODE were dried and degassed at 110 °C under dynamic vacuum and transferred air-free into a nitrogen-filled glovebox. Hexane and Ethanol were purchased from VWR.

Synchrotron X-ray scattering measurements. In situ SAXS experiments were carried out at the X12SA (cSAXS) beamline of the Swiss Light Source at the Paul-Scherrer Institute in Villigen, Switzerland. The wavelength was 16.01 keV and the spot size was approximately 0.4 × 0.3 mm. The exposure time was 0.5 s for each frame. SAXS patterns were collected with a Pilatus 2M detector (254 × 289 mm active area). The sample-to-detector distance was calibrated with a silver behenate standard. A q-range of 0.05 to 7 nm^{-1} was obtained.

Gallium nanoparticle synthesis. The gallium synthesis was carried out according to a protocol adapted from Yarema et al. Visual observations of the synthesis in laboratory conditions are shown in **Figure S1**. For synchrotron experiments, the amounts of reagents were linearly scaled by a factor of 3 in order to cover the X-ray windows. A tailor-made glass reactor equipped with Kapton (polyimide, 50 µm) windows which were held in place with stainless steel, PTFE parts and FFKM O-rings. The beam path was fixed at 8 mm (**Figure S2**). Injection solutions were prepared and filled in a glovebox. They were stored under inert atmosphere in either a glovebox or sealed in air-tight 3-ply polyethylene/nylon bags until use. The reactor was equipped with septa, a stir bar, condenser, and N_2 flushing. The temperature was controlled and recorded with a thermocouple, a remotely operated temperature controller (J-KEM, model 210) and a glass-fiber coated heating band (K. Mösch AG, Switzerland). After flushing the reactor with nitrogen, 21 mL of dried and degassed 1-octadecene were added and the temperature was brought to 280 °C. Patterns of the reactor filled with the solvent were recorded around the growth temperature to be used as a background. To initiate the reaction, the precursor solution ($Ga_2(NMe_2)_6$, the secondary amine (dioctylamine or didodecylamine) and ODE, see **Table S1**) was added with a remote-controlled syringe pump (NE-4000, New Era Pump Systems). The duration of the injection was ca. 10 s. Control experiments were performed in a three-necked flask under conventional Schlenk techniques and by manual precursor injection. Nanoparticles were purified by adding 0.5 mL oleic acid and ethanol in a 1:1 volume ratio to the crude solution. After centrifugation (7000 rpm, 3 min), the precipitates were redispersed in chloroform. The

purification was repeated three times, after which the nanoparticles were stored under ambient conditions until further use.

Table S1: Experimental conditions. Amounts of chemicals used in reactor and in the injection syringe as well as recorded temperature data. T_{inj}: Temperature recorded at t = 0 s, T_{growth}: Average temperature 0 < t < 180 s. DOA: Dioctylamine, DDA: Didodecylamine

	Reactor	Syringe						
No.	1-ODE, ml	$Ga_2(NMe_2)_6$, mg	Amine ID	Amine, ml	[Amine]:[Ga] molar ratio	1-ODE, ml	T_{inj}, °C	T_{growth}, °C
1	21	150	DOA	6.7 ml	30	11.3	277.9	226.1
2	21	150	DOA	6.7 ml	30	11.3	276.9	222.3
3	21	150	DOA	6.7 ml	30	9.8	277.7	240.7
**4**	**21**	**75**	**DOA**	**3.4** ml	**30**	**14.6**	**279.9**	**230.9**
5	21	75	DOA	5 ml	45	13	280.9	227.8
6	21	75	none	-	0	18	280.3	225.5
7	21	75	DDA	2.7 g	21	15.3	280.3	225.5
8	21	75	DOA	1.7 ml	15	16.3	280.1	229.8

Analysis of SAXS data. Azimuthal integration of SAXS 2D images was carried out using a custom MATLAB script. The integrated patterns were further processed using custom Python scripts. To reduce the amount of data and to improve the signal-to-noise ratio, every 5 patterns were averaged, and the corresponding error was obtained by Gaussian error propagation. This yields a time resolution of 2.5 s.

Next, to correct for background scattering by the air, Kapton windows, the reactor and the solvent, SAXS patterns of the reactor filled with octadecene (solvent) were taken during the heating from 200 – 280 °C prior to each experiment (see **Figure S5a,b**). Then the raw SAXS spectra were corrected vs octadecene patterns, averaged between 220 and 240 °C. **Figure S5c,d** plots the uncorrected SAXS patterns of the reactor after injection of the Gallium precursor and the secondary amine and **Figure 2b** of the main text presents the same data after the correction. We also carried out control experiments, heating the reactor with a blank solvent (**Figure S6**), to exclude possible scattering effects coming from chemical instabilities, such as decomposition or polymerization.[1] Note that no transmission correction of the obtained pattern was possible in the measurement mode used. However, the amount of Ga (0.0095 mmol/mL in the final solution) injected will only contribute approx. 3.54 % of the total X-ray total absorption and is constant during the reaction (times > 0 s) and therefore can be neglected.

Fitting of the SAXS curves was carried out using the program SasView 5.0.6[2] using a population-based DREAMS algorithm. A spherical form factor was used:[3]

$$\mathrm{I}(q) = \frac{scale}{V} \cdot \left[3\, V\, \Delta\rho \cdot \frac{sin(qr) - qr\, cos(qr)}{(qr)^3} \right]^2 + background$$

where $scale$ is the volume fraction, V is the volume of the scatterer ($V = \frac{4}{3}\pi r^3$), $\Delta\rho$ is the scattering length density contrast between scatterer and solvent, which was left at the initial standard value of 5, r is the radius of the sphere and $background$ is the background level.

To account for polydispersity, a Schulz distribution was used:[4,5]

$$\mathrm{f}(x) = \frac{1}{Norm}(z+1)^{z+1}(\mathrm{x}/\bar{x})^{\mathrm{z}}\, \frac{exp[-(z+1)x/\bar{x}]}{\bar{x}\Gamma(z+1)}$$

where $\bar{x}$ is the mean of the distribution, $Norm$ is the numerically obtained normalization factor, $\Gamma(\mathrm{x})$ is the gamma function and z is the measure of the width of the distribution:

$$z = (1 - p^2)/p^2$$

where p is the relative polydispersity:

$$p = \sigma / \bar{x}$$

where σ is the root-mean-square deviation from the mean.

This form-factor-only fit was used for all experiments in a region where the contribution due to the structure factor is negligible (i.e. a q values larger than the first correlation peak). These results were used for the kinetic analyses for fair comparison.

For experiments 4 (dioctylamine) and 8 (didodecylamine) an additional hard sphere structure factor was used to investigate the interparticle interaction.[6] To combine the form with the structure factor, the beta decoupling approach was used. The hard sphere model calculates the interparticle structure factor through hard sphere (excluded volume) interactions.[5] It uses the Percus-Yevick closure relationship[7] where the interparticle potential $U(r)$ is:

$$U(r) = \begin{cases} \infty \; for\, r < 2R \\ 0 \; for\, r \geq 2R \end{cases}$$

where r is the distance from the center of the sphere of a radius R. By fitting this model, the effective radius and the volume fraction of the scatterer within the agglomerates are obtained.

The difficulty in the fitting the other data sets with the structure might arise partially due to the larger particles, whose scattering overlap in the q range with the formed agglomerates.

To determine the yield, the scattering patterns were integrated from 0.05 to 2 nm^{-1}:

$$\text{Yield} \propto \int_{0.05}^{2} I(q) \cdot q^2 \; \partial q$$

For the range between 1 and 2 nm^{-1}, the pattern were extrapolated using a Porod function, as shown in **Figure S2**. The yield was normalized from 0 to 100 %.

The number density was calculated according to:[3]

$$number\ density \propto scale/V$$

where $scale$ is the scale factor extracted from the form factor fit and V is the particle volume. The number density was normalized from 0 to 100 %.

Ex situ characterization. Transmission electron microscopy (TEM) was carried out with a Hitachi HT7700 instrument, operated at 100 kV. Fourier-transform infrared spectroscopy was performed on a Bruker Vertex attenuated total reflection setup on a Ge single crystal. The aliquots were dispensed through a 0.2 µm PTFE syringe filter directly on the substrate and measured immediately. Ex-situ SAXS was carried out on a Xeuss 3.0 HR system (Xenocs) equipped with a 2D areal SAXS detector (Eiger 2R 1 M, Dectris) and a Cu K_α microsource. The sample-to-detector distance was 1800 mm and the exposure time was 9 x 600 s in the "virtual detector" mode, making the q-regime larger my moving the 2D SAXS detector to different positions, while keeping the sample-to-detector distance. The data is azimuthally integrated and corrected for transmission. Samples with dioctylamine and 1-octadecene were prepared by filling quartz capillaries under argon and closing them with epoxy glue. The samples were heated in an aluminium block to the desired temperature.

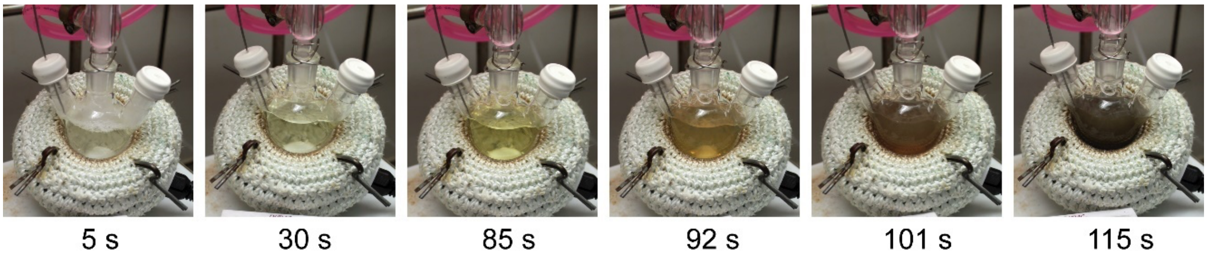

Figure S1: Photos taken during the synthesis of Gallium nanoparticles.

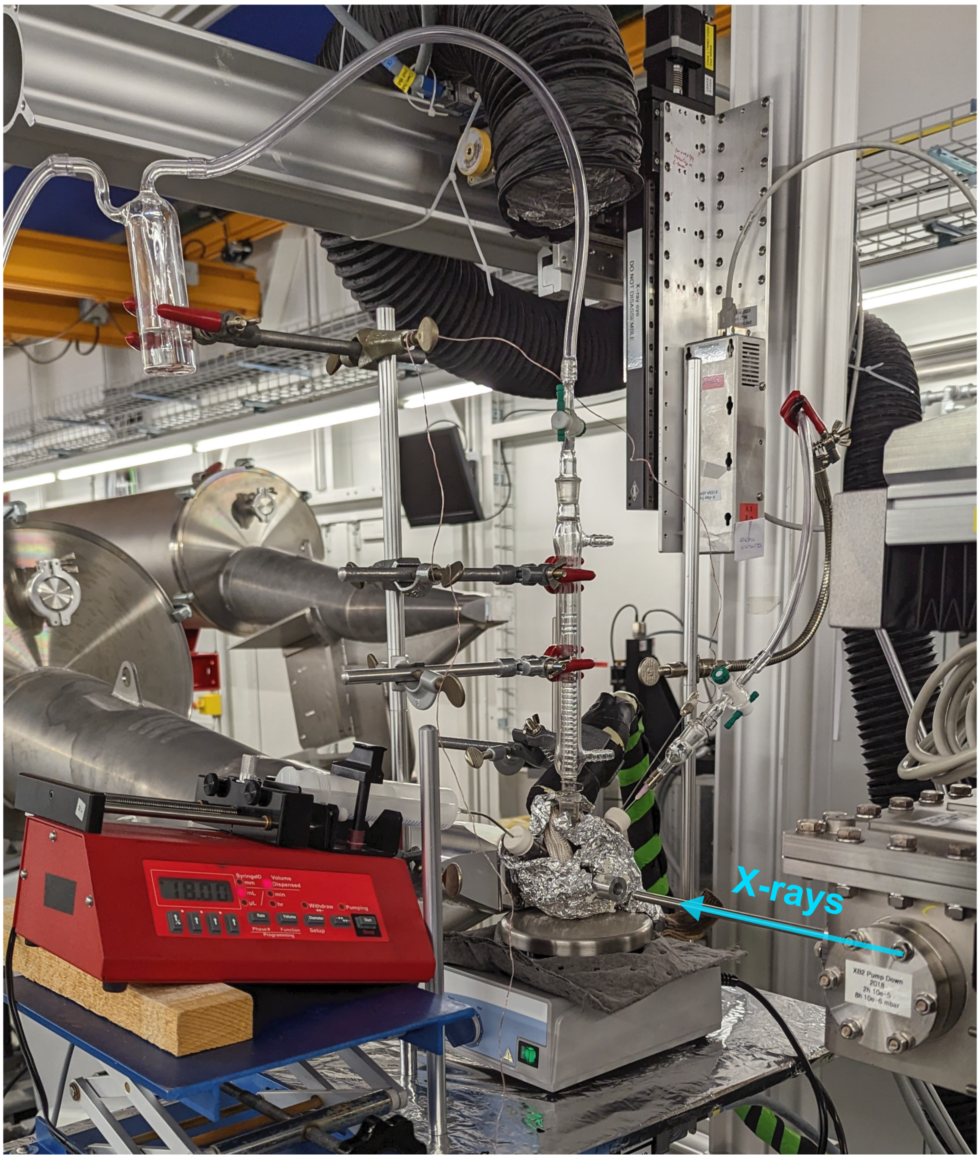

Figure S2: In-situ reactor setup. Glass reactor equipped with stirrer, heating band, thermocouple, nitrogen flushing, condenser and remote-controlled syringe pump.

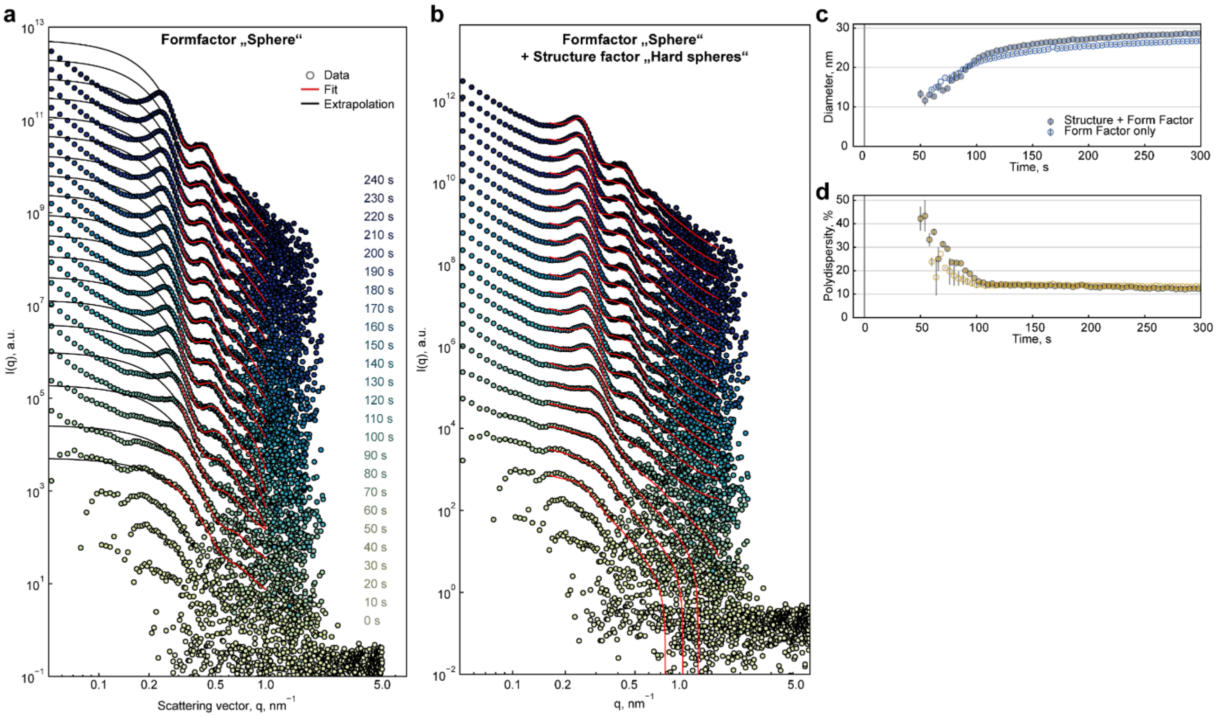

Figure S3: Comparison of fitting without (a) and with (b) hard sphere structure factor for reaction 4 (230 °C, 0.37 mmol Ga, [DOA]:[Ga] = 30). (c) Diameter and (d) polydispersity as function of time for both fitting variants.

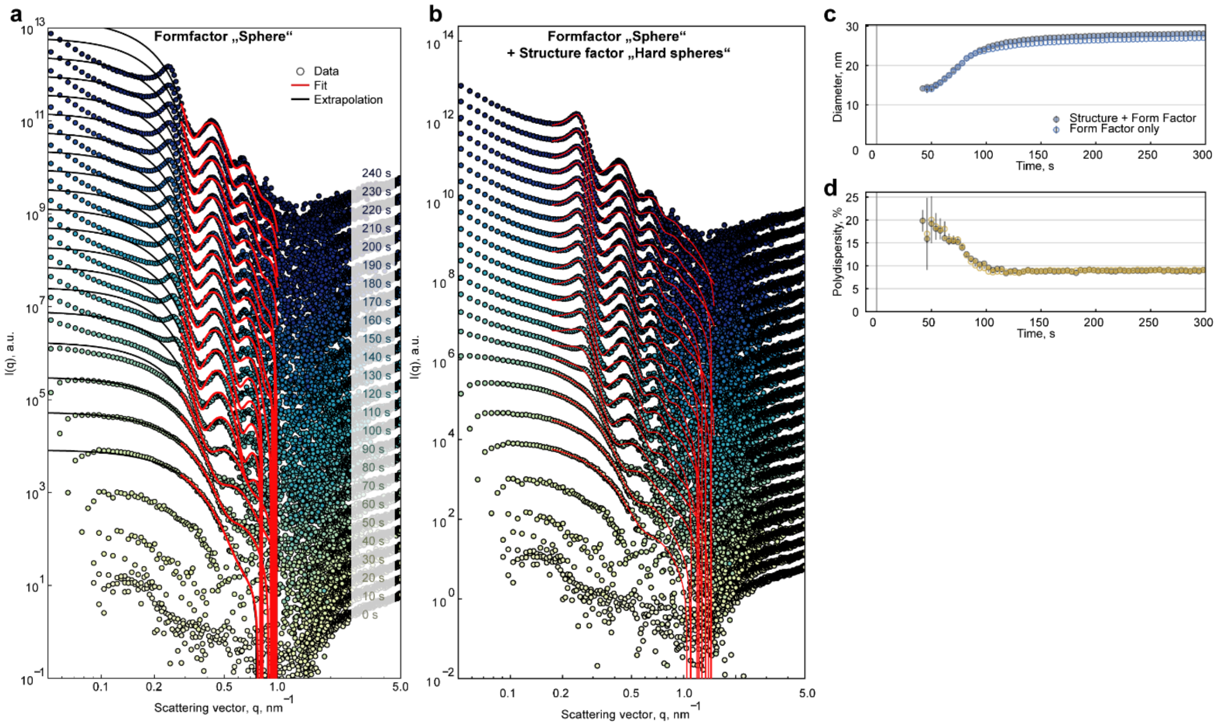

Figure S4: Comparison of fitting without (a) and with (b) hard sphere structure factor for reaction 8 (230 °C, 0.37 mmol Ga, [DDA]:[Ga] = 21). (c) Diameter and (d) polydispersity as function of time for both fitting variants.

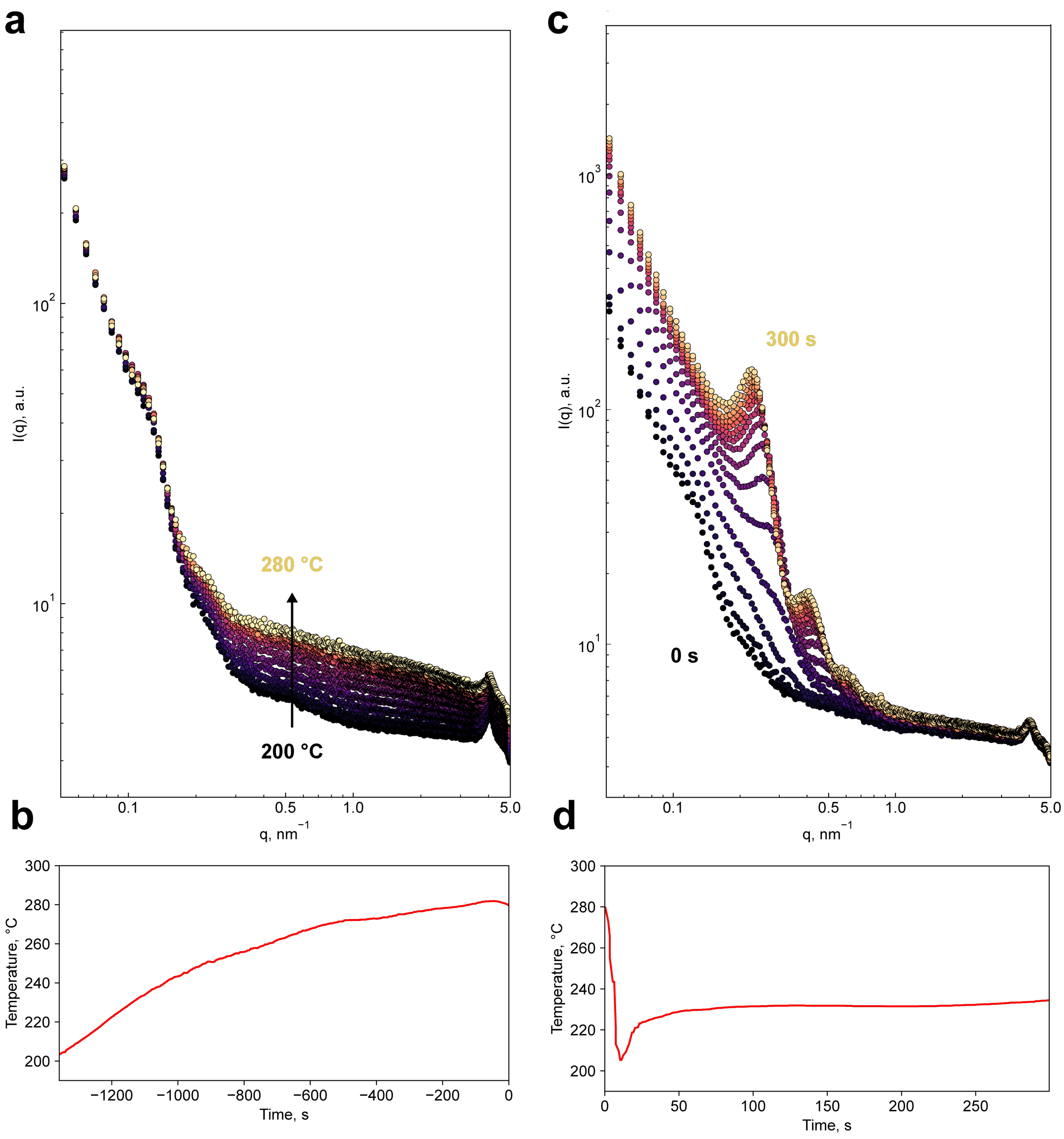

Figure S5: Uncorrected SAXS pattern of the experiment shown in the main Figure 2 (a,b) during heat-up of pure 1-octadecene from 200 to 280 °C and (c,d) in the first 5 minutes after injection of the Gallium precursor and the secondary amine.

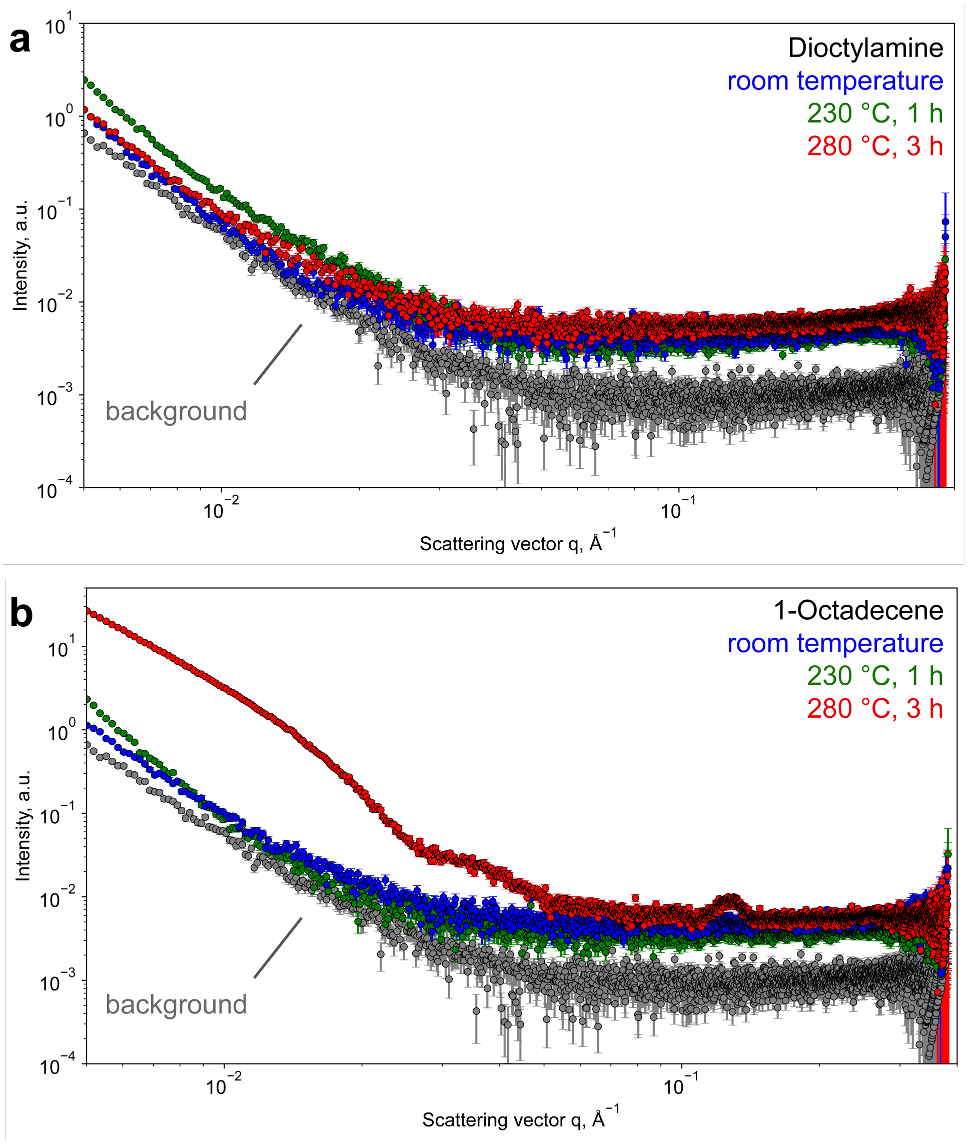

Figure S6: Control ex-situ SAXS experiments for the thermal stability of the solvents. (a) Scattering patterns of capillaries filled with dioctylamine at room temperature (blue), after heating to 230 °C for 1 h (green) and to 280 °C for 3 h (red). For comparison, the scattering pattern of an empty capillary (background, grey) is shown. (b) Scattering pattern of capillaries filled with 1-octadecene at room temperature (blue), after heating to 230 °C for 1 h (green) and to 280 °C for 3 h. For comparison, the scattering pattern of an empty capillary (background, grey) is shown. Even prolonged heating at the growth temperature (230 °C) does not significantly change the scattering pattern. As expected, after heating for 3 h at 280 °C, the octadecene polymerizes,[1] resulting in a significant change of the scattering pattern.

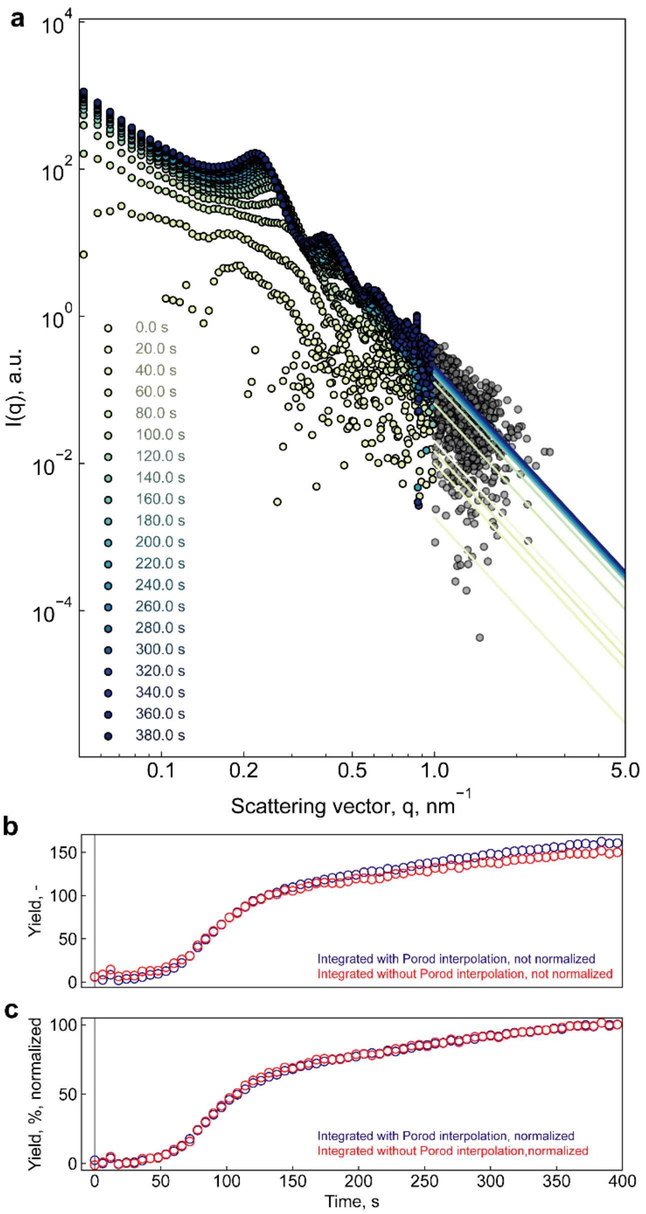

Figure S7: Scattering pattern extrapolation and integration. (a) For determination of the yield, the scattering patterns (circles) were integrated from 0.05 to 2 nm^{-1}. For the range between 1 and 2 nm^{-1}, the patterns were extrapolated using a Porod function ($I(q) = A \cdot q^{-4}$). Note that the patterns were not offset in this figure. (b) Yield with (blue circles) and without (red circles) the Porod interpolation, not normalized. (c) Yield with (blue circles) and without (red circles) the Porod interpolation, normalized to 0 and 100 %.

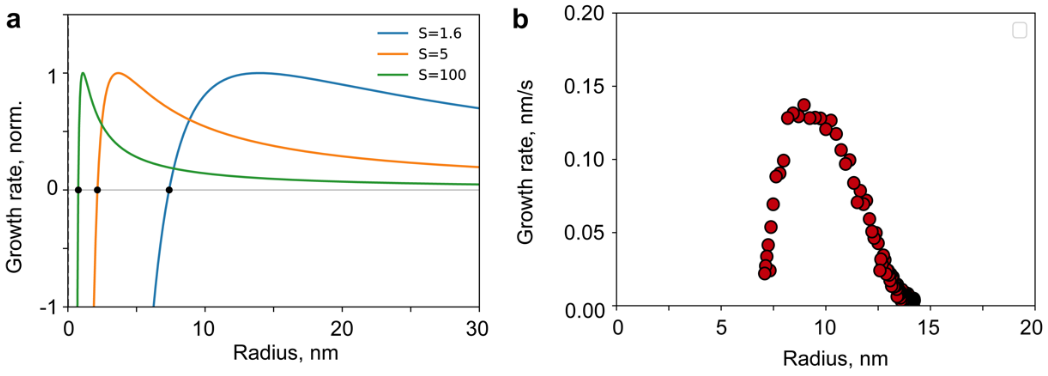

Figure S8: (a) Growth rate as a function of the particle radius for a diffusion-controlled reaction for different supersaturations. Calculated using equations 14/15 from Ref. [8]. Parameters are: $\gamma = 0.725$ J/m^2 (Ref. [9]), $V_m = 10^{-5}$ m^3, $T = 503.15$ K, $\alpha = 0.5$, $D = 10^{-12}$ m^2/s, $k_{g,flat} = 10^5$. (b) Measured growth rate as a function of particle radius for the reaction at $T_{growth} = 230$ °C, [DOA]:[Ga] = 30 and 0.37 mmol Ga.

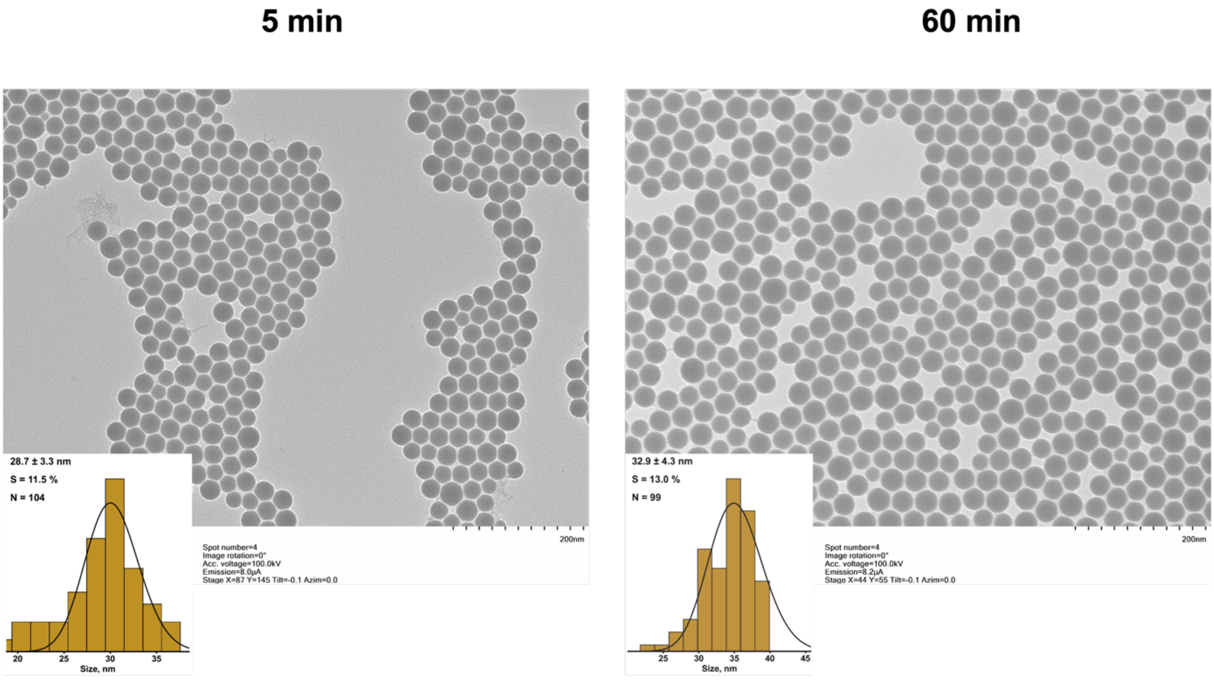

Figure S9: Transmission electron micrographs and size histograms of a control reaction with $T_{growth} = 230$ °C, 0.37 mmol Ga, [DOA]:[Ga] = 30) at 5 min (a), 60 min (b).

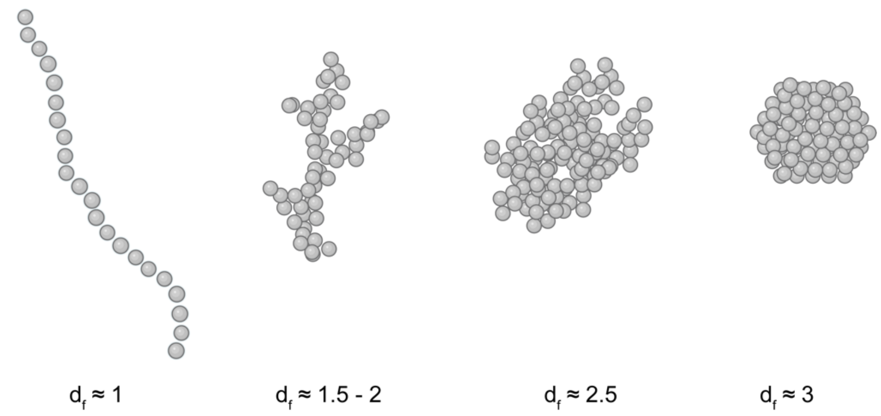

Figure S10: Schematic illustrations of agglomerates with different fractal dimension, ranging from chain-like ($d_f \approx 1$) to dense, spherical agglomerates ($d_f \approx 3$)

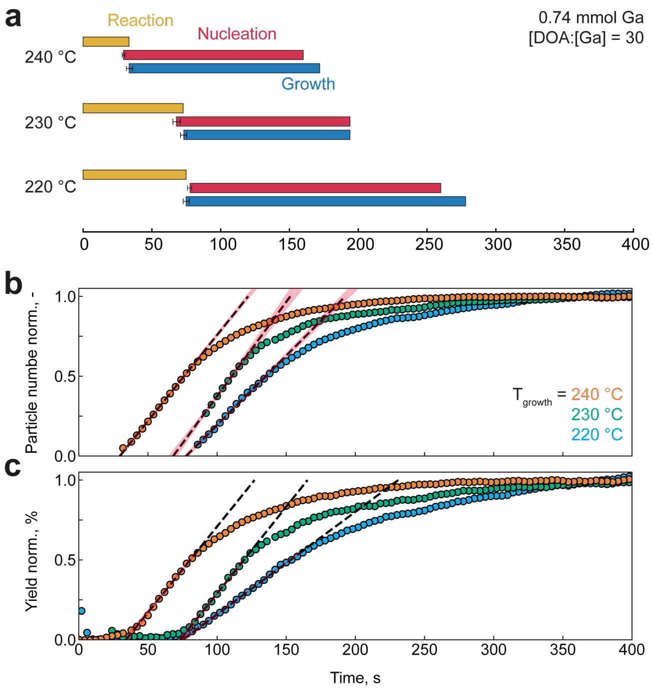

Figure S11. (a) Error bars for the duration of reaction, nucleation, and growth stages of the synthesis, carried out at different temperatures, as presented in Figure 3a of the main text, the are derived from the 95% confidence bands of the linear regression (b,c).

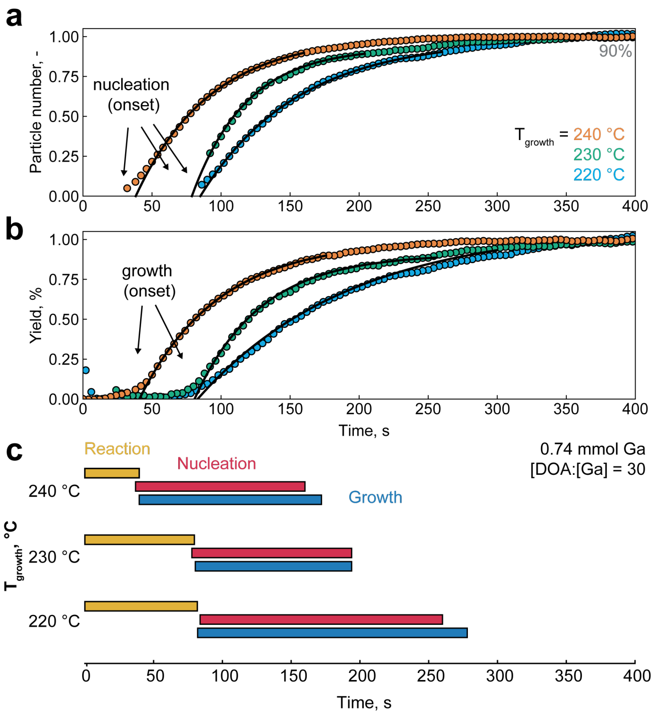

Figure S12: Kinetic model for the determination of the timestamps of reaction, nucleation, and growth stages of the synthesis. Onset of nucleation (a, particle number) and growth (b, yield) is derived from the first order kinetics for the different growth temperatures 220 °C (blue circles), 230 °C (green circles) and 240 °C (red circles). For the first order kinetics the following equation was used: $c(t) = c_{\infty}\,[1 - exp(k\,(t - t_0)]$. Obtained timestamps of the synthesis stages are plotted in (c).

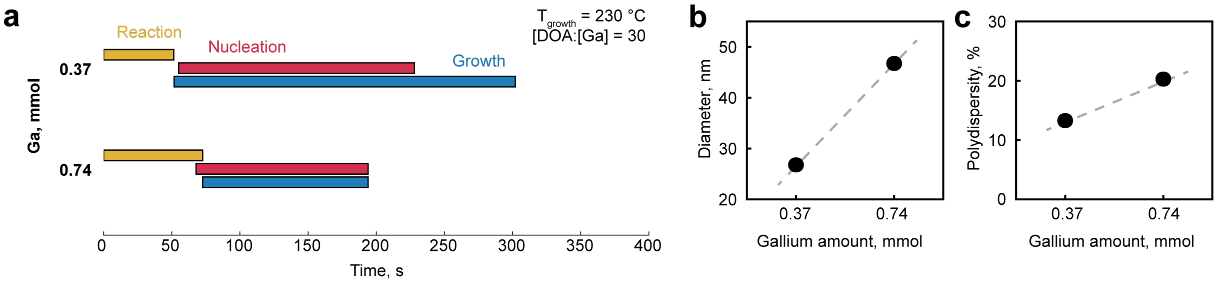

Figure S13: Effect of Ga precursor concentration on kinetics and final particles. From each reaction, characteristic times are extracted for induction (marked by the yield reaching 10 %), nucleation (when the particle number reaches 90 %) and growth (when average particle volume reaches 90 %. (a) Effect of the Ga amount (0.37 or 0.74 mmol), while preserving the growth temperature of 230 °C and a [DOA]:[Ga] ratio of 30. Corresponding steady-state values for (b) size and (c) polydispersity. Grey dashed lines represent a guide to the eye.

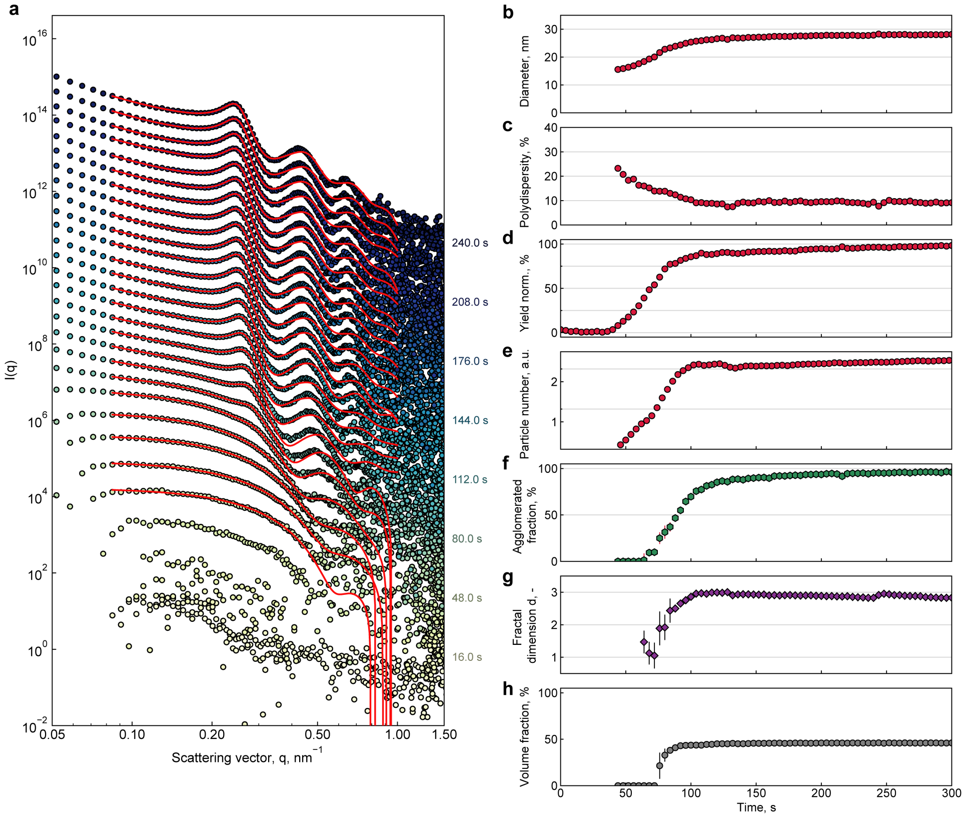

Figure S14: (a) Fitting with an extended model accounting for the ratio of agglomerated particles by linear combination of a spherical form factor and a structure factor, comprising a hard sphere model and a power law applied to the reaction 8 (230 °C, 0.37 mmol Ga, [DDA]:[Ga] = 21). Note that a discussion of the model is given in the section below. Extracted parameters are (b) average diameter, (c) relative polydispersity (Schulz-Zimm distribution), (d) reaction yield, normalized (e) particle number (from form factor fitting only) (f) the fraction of agglomerated particle, (g) the volume fraction of Ga within the agglomerates and (h) the fractal dimension of the agglomerate structures. For clarity, every second data point is shown.

Supplementary Discussion to Figure S14: The fit of the extended model in Figure S10 was adapted from Appel at al.[10]. It combines three models to account for processes in different length scales: Primary particles (spherical form factor, nanometer scale), hard sphere form factor (Interaction of primary particles) and power law (large agglomerates, hundreds of nm).

X-ray scattering intensity is proportional to product of the isotropic form factor $F(q)$ and the structure factor $S(q)$:

$$I(q) \sim \langle |F(q)|^2 \cdot S(q) \rangle$$

For dilute solutions, $S(q) \approx 1$ and the sample can be described using only a form factor. Under considerable interaction of the particles, one must account for the structure factor. To describe a sample consisting of non-agglomerated and agglomerated particles, a linear combination of the form factor and with and without the structure factor is used:

$$I(q) \sim \langle F(q)^2 \rangle \left[\left(1 - X_{agglo}\right) + X_{agglo} \cdot S(q) \right]$$

Where X_{agglo} describes the degree of agglomeration. To describe agglomeration, a hard sphere structure factor is combined with a Power law to describe the features a low q:

$$S(q) = S_{HS}(q) + c \cdot q^{-d}$$

where d is associated to the fractal dimension of the agglomerates and c, a scaling factor. Due to the high number of parameters (8), a stable, meaningful and reliable fit was only possible for reaction 8, where there is a distinct transition between mainly non-agglomerated particles and later agglomerated ones.

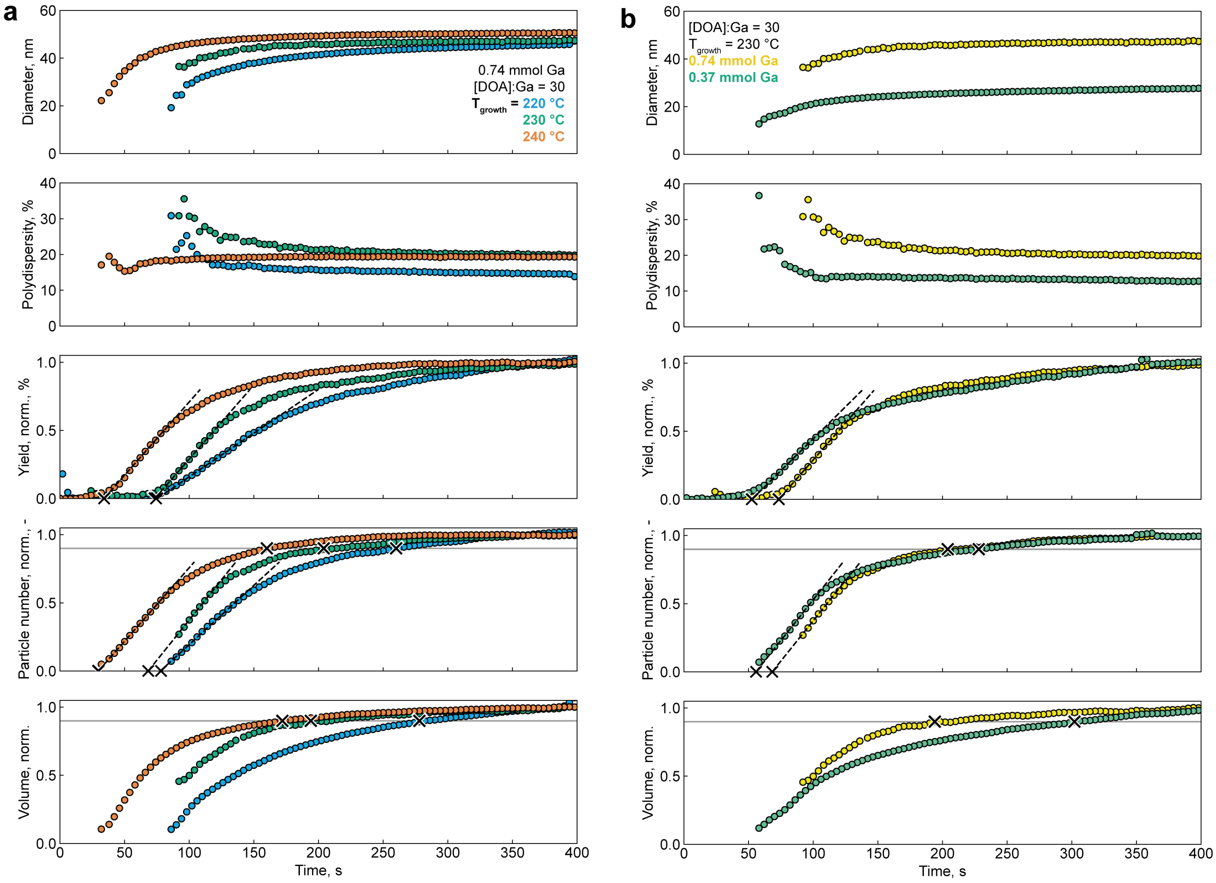

Figure S15: Fit results from the form factor only fitting and analysis of characteristic times for (a) varying T_{growth}: 220 °C, blue circles), 230 °C (green circles) and 240 °C (orange) circles) (b) varying Ga precursor amounts: 0.37 mmol (green circles) and 0.74 mmol (yellow circles) and

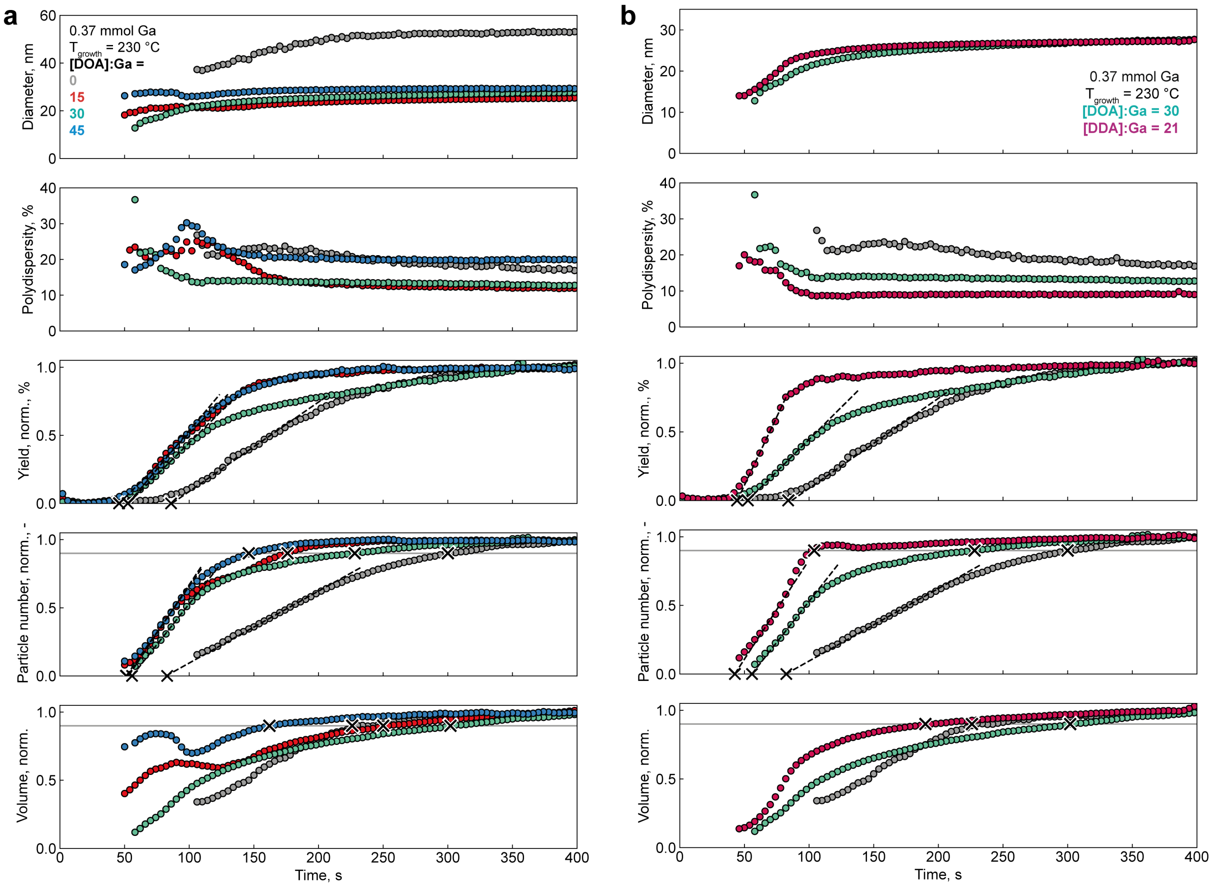

Figure S16: Fit results from the form factor only fitting and analysis of characteristic times for (a) [Ga[:[DOA ratios: 0 (grey), 15 (red), 30 (green) and 45 (blue) and (b) different amines: Dioctylamine (green) and didodecylamine (purple). Note that a slightly lower amount of DDA was used due to limited solubility of the amine in octadecene.

Table S2: Characteristic times and kinetics analysis for all reactions

Reaction	Yield (onset), s	Nucleation (onset), s	Nucleation (end), s	Growth (end), s	Nucleation rate, %/s	Growth rate, %
1	73	68	194	194	1.18	1.09
2	75	78	260	278	0.87	0.64
3	34	30	160	172	1.08	1.08
4	53	56	228	302	1.20	0.94
5	53	51	146	162	1.35	1.13
6	84	82	226	226	0.53	0.66
7	44	43	104	190	1.44	1.99
8	46	53	176	250	1.43	0.95

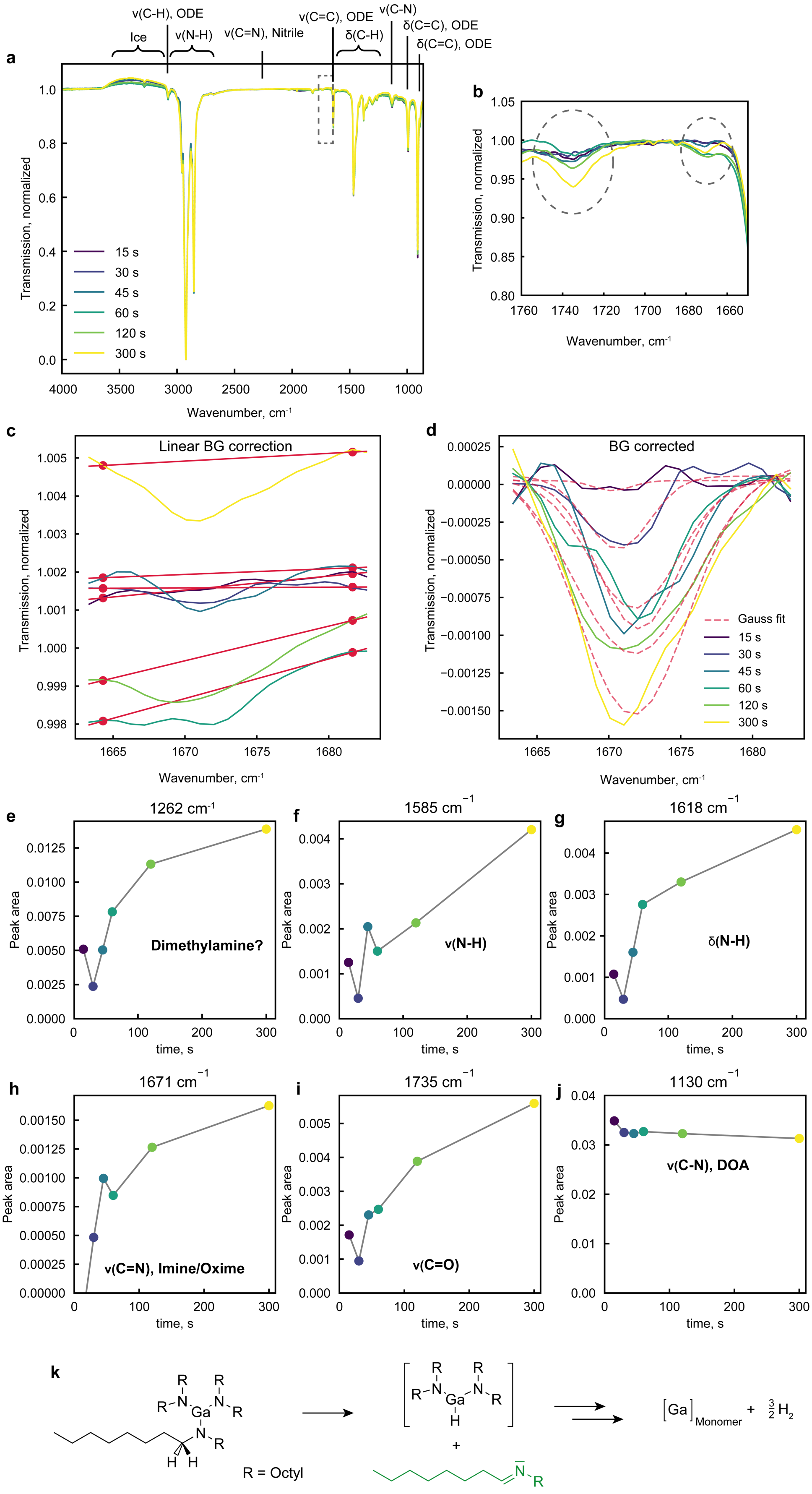

Figure S17: FTIR analysis of aliquots taken during Ga synthesis using 0.37 mmol Ga, a [DOA]:[Ga] ratio of 30 and a T_{growth} of 230 °C. (a) Overview spectra of the reaction solution taken at 15, 30, 45, 60, 120 and 300 s. The

spectra were normalized. Possible assignments of the bands are indicated. While no stark changes are apparent here, small changes can be observed upon zooming in (b), such as in the region between 1740 and 1665 cm^{-1}. (c) For background correction, a linear background (red lines) is subtracted. To extract the peak areas, Gaussian functions (dotted red lines) were fitted to the data. Five bands that undergo slight changes were identified and the peak areas plotted as function of time: (e) 1262 cm^{-1}, (f) 1585 cm^{-1}, (g) 1618 cm^{-1}, (h) 1671 cm^{-1}, and (i) 1735 cm^{-1}. For comparison, the area of the C-N bending mode of dioctylamine (1130 cm^{-1}) is shown, which only slightly decreases, given the large amine excess. The band at 1671 cm^{-1} fits nicely the literature values for alkyl-substitutes aldimines[11]. Therefore, one pathway of the reduction of the gallium-dialkylamine complex to metallic Ga could be via oxidation on the secondary amine to an imine. The gallium-hydride complex is shown, but given the instability of gallium hydride (decomposition at > - 20°C)[12] it represents a short-lived intermediate at most. Note that amine oxidation to imines and alidimines was shown before in CoO_x nanocrystal synthesis.[13] The other bands might correspond to other side products, perhaps with a amine group, such as dimethylamine (1262 cm^{-1}) or oxidation products (1735 cm^{-1}) due to short air exposure prior to the measurement.

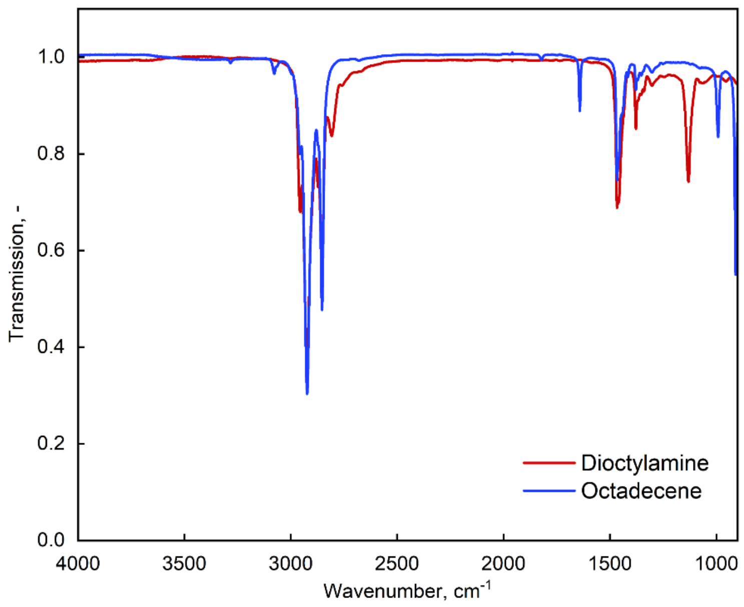

Figure S18: FTIR reference spectra of (a) pure dioctylamine (red line) and (b) pure octadecene (blue line).

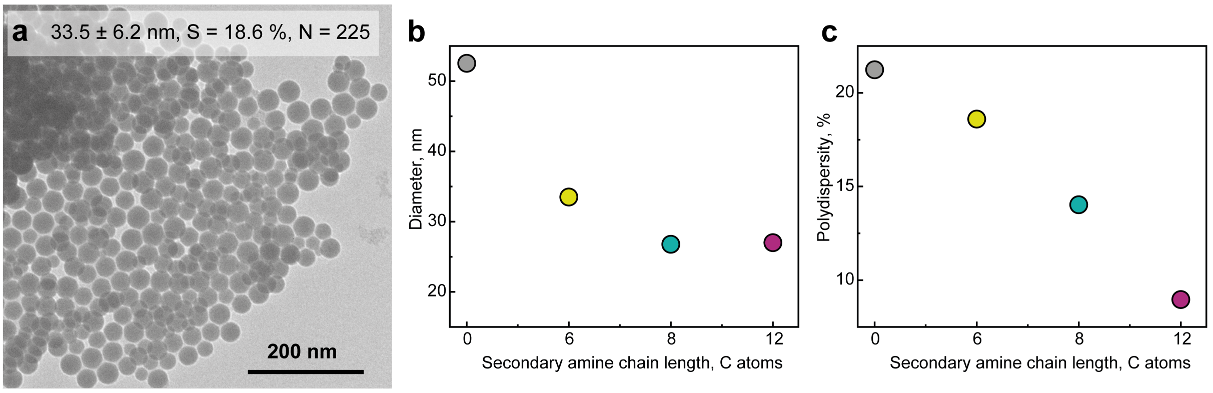

Figure S19: (a) Transmission electron micrograph of control reaction with dihexylamine (reaction conditions: T_{growth} = 230 °C, 0.37 mmol Ga, [Dihexylamine]:[Ga] = 30, T_{growth} = 5 min). The (a) size (34 nm) and (b) size distribution (19 %) of Gallium nanoparticles synthesized using dihexylamine fall in the trend between dioctylamine and no amine.

Supplementary References:

1. Dhaene, E.; Billet, J.; Bennett, E.; Van Driessche, I.; De Roo, J. The Trouble with ODE: Polymerization during Nanocrystal Synthesis. *Nano Lett.* **2019**, *19* (10), 7411–7417.
2. *Sasview 5.0.6.*
3. *https://www.sasview.org/docs/user/models/sphere.html.*
4. *Schulz Distribution — SasView 6.0.0 documentation.*
5. Kotlarchyk, M.; Chen, S. Analysis of Small Angle Neutron Scattering Spectra from Polydisperse Interacting Colloids. *The Journal of Chemical Physics* **1983**, *79* (5), 2461–2469.
6. *hardsphere — SasView 6.0.0 documentation.*
7. Percus, J. K.; Yevick, G. J. Analysis of Classical Statistical Mechanics by Means of Collective Coordinates. *Phys. Rev.* **1958**, *110* (1), 1–13.
8. Talapin, D. V.; Rogach, A. L.; Haase, M.; Weller, H. Evolution of an Ensemble of Nanoparticles in a Colloidal Solution: Theoretical Study. *J. Phys. Chem. B* **2001**, *105* (49), 12278–12285.
9. Hardy, S. C. The Surface Tension of Liquid Gallium. *Journal of Crystal Growth* **1985**, *71* (3), 602–606.
10. Appel, C.; Kuttich, B.; Kraus, T.; Stühn, B. In Situ Investigation of Temperature Induced Agglomeration in Non-Polar Magnetic Nanoparticle Dispersions by Small Angle X-Ray Scattering. *Nanoscale* **2021**, *13* (14), 6916–6920.
11. Parry, K. A. W.; Robinson, P. J.; Sainsbury, P. J.; Waller, M. J. Nuclear Magnetic Resonance and Infrared Spectra of Some Aldimines (Azomethines). *J. Chem. Soc., B:* **1970**, 700.
12. Downs, A. J.; Pulham, C. R. The Hydrides of Aluminium, Gallium, Indium, and Thallium: A Re-Evaluation. *Chem. Soc. Rev.* **1994**, *23* (3), 175.
13. Calcabrini, M.; Van den Eynden, D.; Ribot, S. S.; Pokratath, R.; Llorca, J.; De Roo, J.; Ibáñez, M. Ligand Conversion in Nanocrystal Synthesis: The Oxidation of Alkylamines to Fatty Acids by Nitrate. *JACS Au* **2021**, *1* (11), 1898–1903.
